# Sprayer boom height measurement in wheat field using ultrasonic sensor: An exploratory study

**DOI:** 10.3389/fpls.2022.1008122

**Published:** 2022-11-22

**Authors:** Xueguan Zhao, Changyuan Zhai, Songlin Wang, Hanjie Dou, Shuo Yang, Xiu Wang, Lipin Chen

**Affiliations:** ^1^ Intelligent Equipment Research Center, Beijing Academy of Agriculture and Forestry Sciences, Beijing, China; ^2^ National Engineering Research Center for Information Technology in Agriculture, Beijing, China; ^3^ National Engineering Research Center of Intelligent Equipment for Agriculture, Beijing, China; ^4^ College of Mechanical Engineering and Automation, Liaoning University of Technology, Huludao, China

**Keywords:** boom height control, wheat canopy detection, ultrasonic detection, boom sprayer, precision agriculture

## Abstract

In order to explore the influencing factors and laws of ultrasonic sensor detecting wheat canopy height, designed an ultrasonic sensor detection height test platform with speed adjustable function. Taking step surface, bare soil and wheat canopy as the research objects, a canopy height calculation method based on K-mean clustering is proposed, and the response characteristics of ultrasonic detection to three media under different operating speeds are explored. Firstly, the step detection test results show that the average detection error of ultrasonic sensor is 1.35%. When the sensor detection distance is switched at the step, with the increase of detection distance, the actual offset at the step increases first and then tends to be stable, and the maximum offset is 10.4cm. The test results of bare soil slope show that the relative error between the detection distance and the manual measurement distance is 1.4% under quasi-static conditions. The leading or lagging of detection under moving conditions is affected by multiple factors such as terrain undulation, speed and detection range. The detection test results of wheat canopy showed that the detection distance was larger than the manual measurement distance, and the smaller the canopy density, the greater the detection error and error variance. When the moving speed is 0.3m/s-1.2m/s, the average detection deviation of the ultrasonic sensor for five kinds of wheat canopy density is 0.14m, and the maximum variance of the detection deviation is 0.07cm2. In this paper, the research on the response characteristics of ultrasonic to the detection of bare soil and sparse canopy in wheat field can provide technical support for the detection of crop canopy in the field.

## 1 Introduction

**Figure 1:**
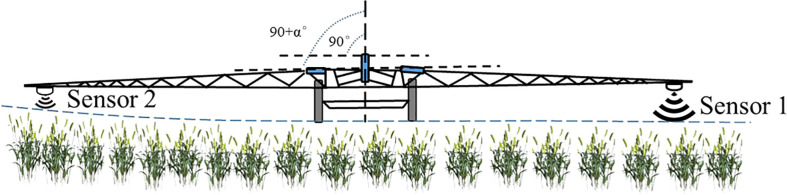
Field crop canopy detection based on ultrasound. For a large spray boom, even a small tilt may result in one end of the spray boom coming very close to the ground or crop canopy.

With the moderate promotion of agriculture on a large scale in China, large-scale farmland is gradually being formed. Boom sprayer, with its advantages of wide spraying width and high operational efficiency, is being used more and more widely in production (Zheng and Xu, 2021). When working in the field, localized unevenness in the plot can cause large undulations at the end of the spray boom (Drocas et al., 2009; Tahmasebi et al., 2012). For sprayers with larger spray boom, even a small tilt may result in one end of the spray boom being high above the ground or crop canopy (Jeon et al., 2004) (as shown in **Figure 1**), with serious droplet drift. The other end of the spray boom is closer to the ground or crop canopy, and in severe cases the end of the spray boom can touch the ground or vegetation canopy, causing damage to the spray boom or vegetation. It is therefore essential to achieve a reasonable and stable spray height between the nozzle and the spray target, with the spray boom in a parallel attitude to the ground (Gil et al., 2015; Herbst et al., 2015). Spray boom self-balance control technology is an important way of maintaining a stable attitude of the spray boom relative to the ground, and spray boom tilt monitoring is a prerequisite for spray boom self-regulation (Wei et al., 2015; Cui et al., 2017).

The height of the spray boom at different lateral positions, as an important indicator parameter of the spray boom attitude, is the main influencing factor on the quality of the spray distribution (Ramon and Baerdemaeker, 1997; Ramon et al., 1997a; Ooms et al., 2003). The accuracy of the spray boom height detection directly affects the response characteristics of the spray boom height control system (Dou et al. 2021a). Researchers at domestic and international level have conducted research on different sensing detection methods for detecting spray boom tilt heights and have also made considerable progress. Different detection methods have their own advantages and disadvantages, and adapt to different application scenarios (Andrade et al., 2014; Barker et al., 2016). The sensors used in the detection methods mainly include tilt sensor, acceleration sensor, laser sensor, contact sensor, ultrasonic sensor, etc. Their advantages and disadvantages are as follows:

- Inclinometer, which converts the monitored displacement information of the spray bar into angle information (Cui et al., 2019a; Cui et al., 2019b). To ensure the test accuracy, it is necessary to calibrate the linear relationship between the A/D value of the test system and the input Angle θ, and the installation and calibration accuracy are required to be high.
- Acceleration sensor, through the analysis of the signal time-domain waveform, obtain the change curves of the excitation signal and response signal with the excitation time, mainly to solve the problem that the vibration of the spray bar of the traditional spray bar sprayer affects the spray quality (Herbst et al., 2015; Wang et al., 2019).
- Laser sensor, in the horizontal installation mode, the laser sensor emits light to a fixed laser receiving plate, and the displacement change of the spray boom in the field movement process is measured by marking the position of the laser point on the receiving plate (Ooms, 2002). Laser scatterability is poor, and it is difficult to provide sufficient data for cases like wheat with low canopy density and irregular surface structure (Ehlert et al., 2009).
- Contact sensor, the height of the spray boom is detected through by elastic deformation of the contact rod of the sensor. Although the detection method is simple and convenient, it has the risk of damaging crops, which limits its application scope.
- Ultrasonic sensors, Ultrasonic detection is one of the most mature distance measurement technology at present (Llorens et al., 2021). Ultrasonic has the advantages of high frequency, good directivity and high accuracy, and is widely used in the agricultural field. They are cheap, simple in information processing, convenient in installation, and economical and applicable, so they are commonly used as a ranging method in agriculture.

pUltrasonic detection of canopy distance and density information was applied to fruit tree canopy detection (Brown et al., 2008; Maghsoudi et al, 2015; Liu et al., 2016) to detect the presence of canopy for the purpose of target application. In field production, crop height, cover and biomass density are very important parameters for assessing crop stands. Ultrasonic distance sensors were already used by numerous researchers to measure plant height (Chang et al., 2017). Sui used ultrasonic sensors for estimating cotton height (Sui et al., 2012a; Sui et al., 2012b; Sharma and Ritchie, 2015). Chang developed a system for measuring the height of wild blueberry plants based on ultrasonic (Chang et al., 2017). The findings provide a basis for height detection in field crops, but it is difficult to extend to other crops due to the differences in canopy structure between crops. Those spray boom height detection systems performed relatively well on flat bare ground (Dou et al. 2021b), with larger leaf canopies (cotton, maize). The suitability and effectiveness of detection for sparse canopies in wheat is unclear. The data detected in sparse environments may be on the surface of the wheat canopy, on the ground, on the leaves at any height of the plant, or even anomalous data. Therefore, these signals need to be separated from the canopy surface signals, otherwise the distance value data extracted to the canopy is less accurate. Ultrasonic sensors are susceptible to jumps in the process of detecting canopy height due to interference from external factors, which affects detection accuracy. The applications of ultrasonic sensors in field crop characterization have all focused on studies comparing manual estimates, other sensors and results obtained using ultrasonic sensors (Llorens et al., 2011; Zhou et al., 2021). Information on the interaction between sound waves and the canopy and how it interferes with ultrasonic sensor estimates has not been mentioned. The aim of this study was to explore the response characteristics of ultrasonic waves to the detection of bare soil in the field and sparse canopy of wheat fields under different operating speeds so as to evaluate the detection performance of ultrasonic sensors. Based on ultrasonic sensing detection technology, a spray boom height detection platform was designed to simulate field movement of spray booms. Furthermore, the dynamic detection characteristics of the ultrasonic sensors for different detection targets were investigated using regular steps, the field surface and the wheat canopy as detection targets, providing a technical basis for the research of spray boom height detection methods and spray boom autonomous balance control systems.

The research is divided into five parts as follows: The first section introduces the previous work and the purpose of the research. The second section gives the design of the ultrasonic sensing detection platform and the experiments on the detection performance of the LIDAR sensor. The third section presents the results of the detection field tests. The fourth section discusses the test results. The fifth section provides conclusions.

## 2 Materials and methods

### 2.1 Design of test platform

**Figure 2:**
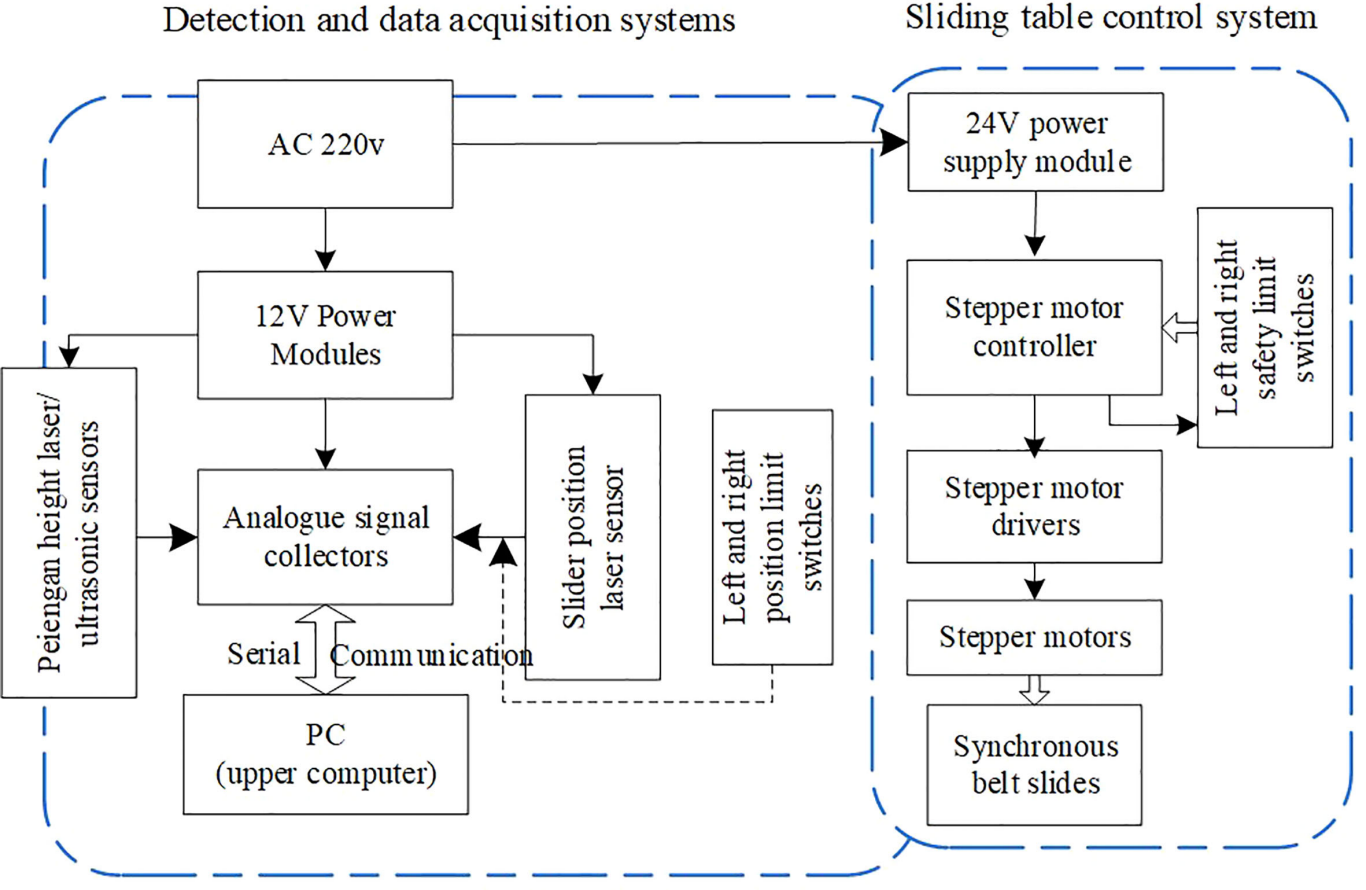
Block diagram of boom height probing platform. The test platform for ultrasonic sensor to detect the height of spray boom mainly includes sliding table unit and ultrasonic sensor detection unit, which can meet the static and dynamic ultrasonic detection test requirements.

In order to explore the performance of the canopy height detection method based on ultrasonic sensing technology, an ultrasonic sensor with adjustable speed function was built to detect the height of the spray rod. The platform mainly includes a sliding table unit and an ultrasonic sensing detection unit. The timing belt sliding table unit mainly includes stepper motors, drives, controllers, and safety limit switches. The operating speed is set by the controller, and the speed of the stepper motor is adjusted, and the slide is moved at the set speed to achieve the simulated spray pole field movement scene. The safety limit switch protects the slider from hitting both ends of the sliding track. The laser alignment correction unit includes a laser emitter and a retro reflection correction plate to correct the linear motion of the slide and provide a stable test system basis for further detection tests. The ultrasonic sensing detection unit mainly includes ultrasonic sensors, signal collectors, and spray rod height detection systems to realize the real-time acquisition and preservation of ultrasonic detection information. The detection block diagram of the test bench is shown in **Figure 2**.

#### 2.1.1 Design of movable mechanism

**Figure 3:**
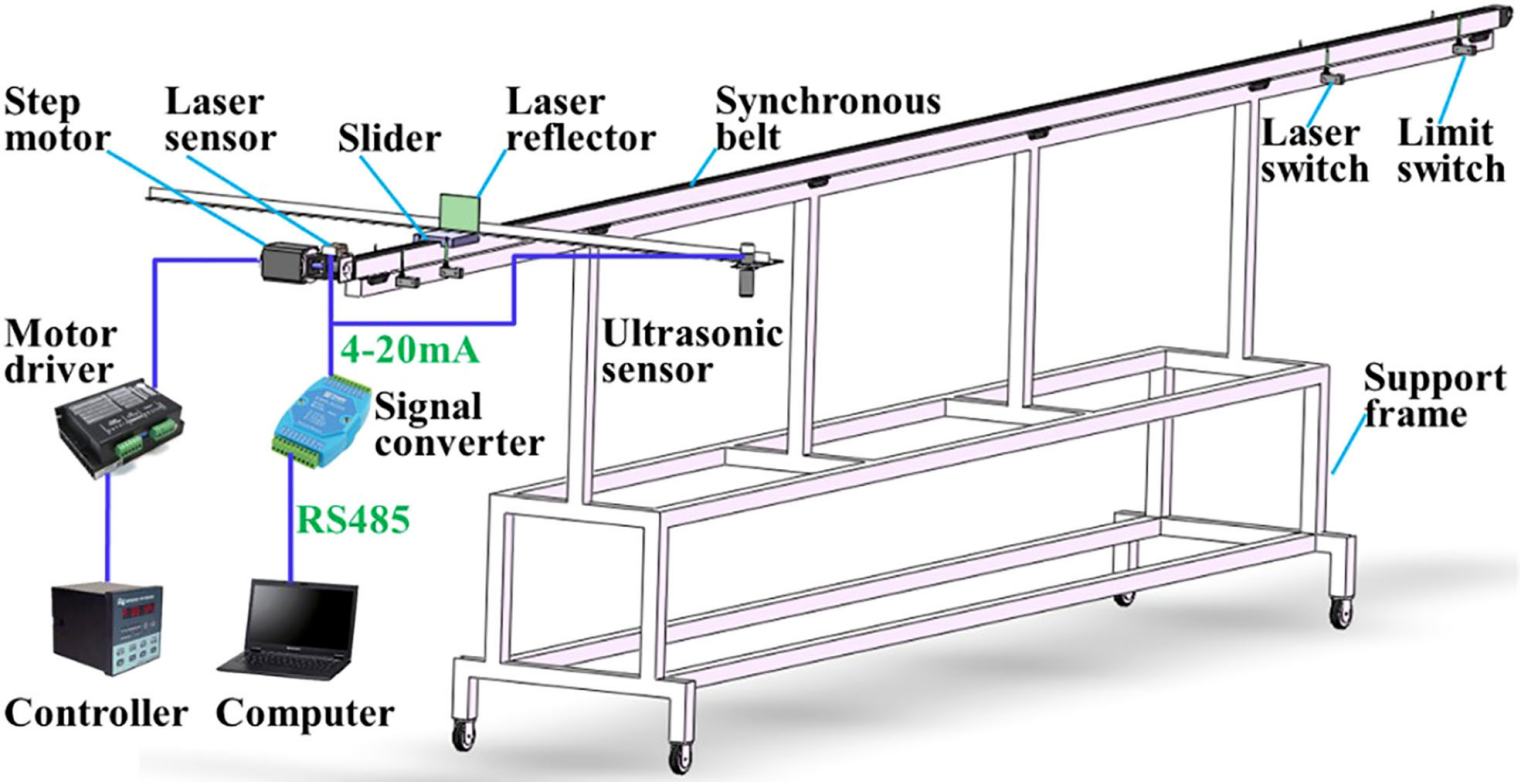
Structural diagram of boom height probing platform based on laser/ultrasonic sensor. The designed effective length of sliding platform track is 6.0 m, which can realize the test under different speed conditions.

The effective length of the sliding platform track of the design is 6.0 m, as shown in **Figure 3**. The sliding track is bridge-mounted, with a removable slider block measuring 0.3 m long for mounting the fixed sensor mounting rod. The stepper motor is selected from the 86HB250-80B stepper motor produced by Shantou Hongbaoda Electromechanical Co., Ltd., with a rated voltage of 24V and a torque of 8.5N· M, the maximum acceleration of the movable sliding table is 13.2 m/s2. The stepper motor driver communicates with the controller via the RS485 serial port (KH-01 Shenzhen Yixing Technology Co., Ltd.) and is supplied with AC220 V. There are 4 limit switches at both ends of the sliding track (CZ-7166Detu Instrument (Shenzhen) Co., Ltd.). Among them, the two outer limit switches are used as safety limit switches to prevent the slider from hitting both ends of the sliding track, and the two inner limit switches are used as position limit switches, limiting the motion distance of the slide to 5.0 m as the moving stroke of the sensor.

Spray rod height ultrasonic sensor (WUB2000-30GM75-1-V15, Guangzhou Weiheng Electronics Corporation, China) detection range 0.2-2m, blind zone 0-0.1m, acquisition frequency 30Hz, 4-20mA current output, supply voltage of 12 V, resolution 0.52mm, with temperature compensation function, temperature drift %1.5%. The laser sensor for slider position determination (ALM80201 Shenzhen Shenpu Electric Co., Ltd.) has a ranging range of 80.0 m and outputs a current signal of 4-20 mA. The data acquisition card (analog acquisition module 4-20mA current, Bominte Chengdu Technology Co., Ltd.) simultaneously collects the measurement data of the ultrasonic sensor and the laser sensor, communicates with the host computer through the 485 serial port, and stores the collected test data into the database. The host computer software adopts independent design to develop a universal acquisition system for analog signals, realizes the communication between the computer and the data acquisition card, and controls the acquisition and storage of test data.

#### 2.1.2 Software design of test platform

**Figure 4:**
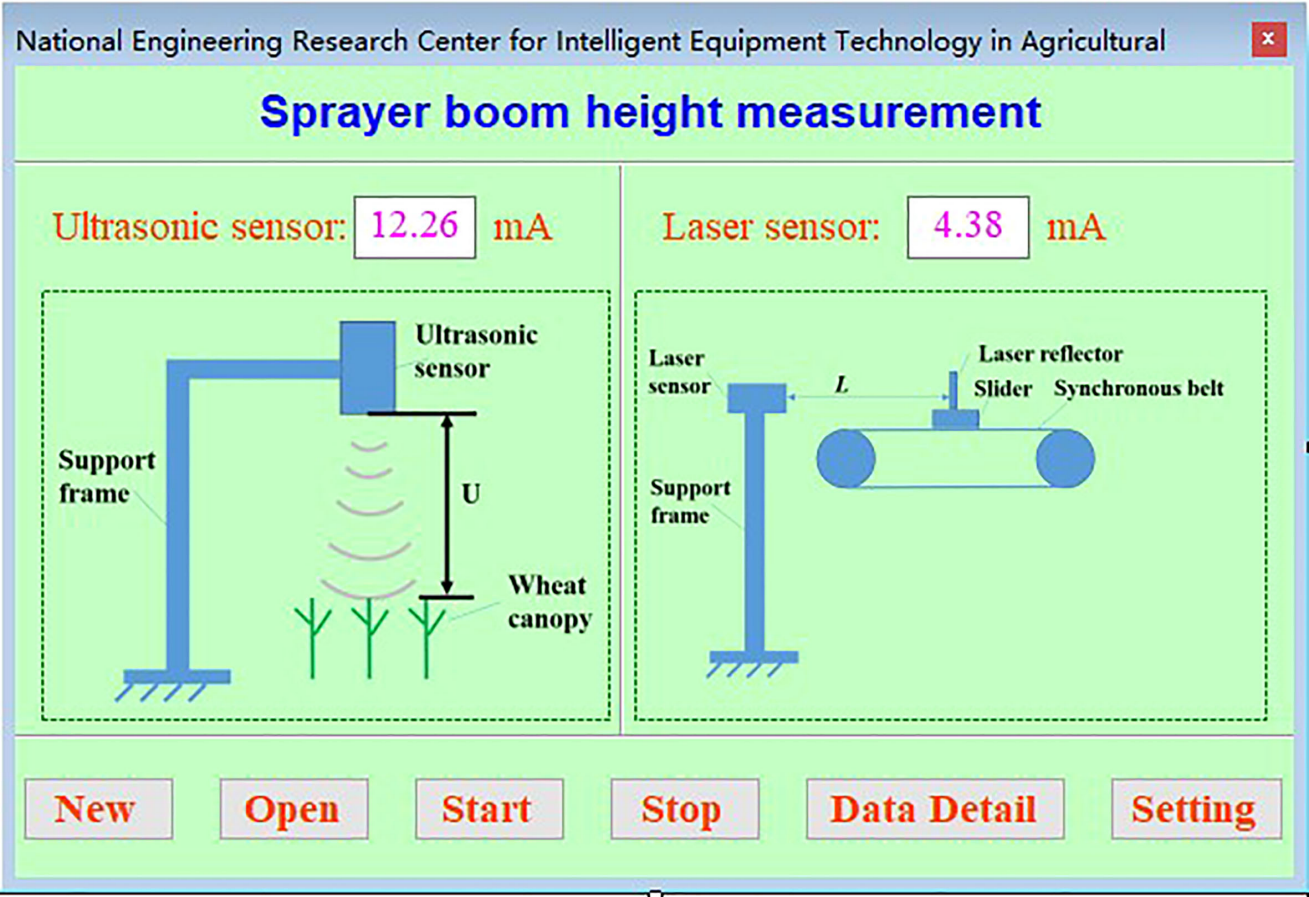
Ultrasonic detection system control interface. The spray boom height detection and test system based on ultrasonic sensor can realize test control, real-time information display and data storage.

The interface of the spray rod height detection test system based on ultrasonic sensing is shown in **Figure 4**, which mainly includes the real-time display of ultrasonic sensors, laser ranging sensors, working status monitoring display, test control, data saving and setting functions. Access database is used, the database header information includes serial number, time, ultrasonic sensing detection height information, laser measurement distance information. The parameter setting mainly includes the movement speed and distance of the sliding platform. When working, first create a new test, open the communication port, and then click start button system to start working, slide the table according to the set speed and distance to drive the ultrasonic sensor movement, the height information obtained and the distance information of the current movement are saved to the database in real time, and the saving interval is 0.5s.

### 2.2 Calibration of ultrasonic sensor

**Figure 5:**
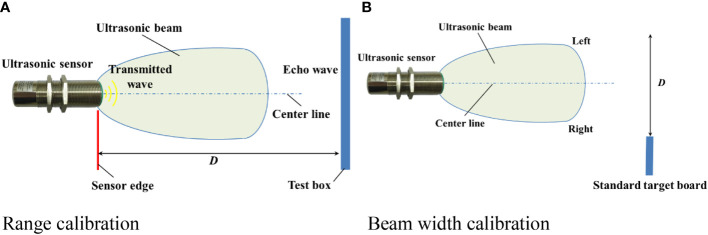
Schematic diagram of ultrasonic sensor beam width calibration. Place the standard reflector plate perpendicular to the ultrasonic emission direction, and then move it vertically from the center to both sides. Calibrate the detection range through the test. **(A)** Range calibration **(B)** Beam width calibration.

The information on the detection distance is given as an output in the form of an electrical analogue signal. In order to obtain a relationship between the ultrasonic sensor output current and the actual detection distance, a detection distance calibration was performed as shown in **Figure 5**. The standard plate (100mm* 100mm) being detected was placed in the direction of the vertical beam emission. The actual distance between the transducer and the reflector plate was determined by means of a tape measure and the data acquisition card collected the current signal from the ultrasonic transducer. The output current value was read directly from the display on the acquisition card and was repeated three times for each distance, averaged and recorded. The output current of the transducer was taken as the horizontal coordinate and the actual distance of the transducer was taken as the vertical coordinate and the detection data was linearly fitted.

Ultrasonic sensors are capable of performing reliable measurements in a specific area based on ultrasound. The detection range of an ultrasonic sensor can be divided into the working detection range, the limit detection range and the blind zone. The size of the acoustic cone angle is indicated in the specifications of the ultrasonic transducer, which makes it simple to determine the range of the transducer’s acoustic cone. However, due to the irregular distribution of the acoustic cone, it is difficult to obtain an accurate and effective working detection range of the transducer from the cone angle alone and the working detection range needs to be calibrated by test. The standard reflector plate is placed perpendicular to the direction of ultrasonic emission and then moved in a vertical direction from the center to the sides, observing the current signal output from the transducer on the acquisition card in real time during the movement. When the reflector plate moves to a position where it cannot be detected, the distance between the edge of the reflector plate and the center line of the sensor is measured, and the distance is the unilateral sound cone width of the ultrasonic sensor. The cone width calibration test selects a calibration detection distance at 0.1-1.9m intervals within a detection distance of 0.1m. 19 calibration detection distances are selected, and each calibration detection distance is repeated three times for the average and the data is recorded to obtain the left and right cone widths of the ultrasonic transducer respectively. The left and right cone widths were summed to obtain the exact cone width at each calibration distance.

### 2.3 Clustering algorithm

The detection data of the wheat canopy by ultrasonic sensors need to be further processed to separate the canopy data and non-canopy data. Before clustering the data, the abnormal burr data are screened by sliding window filtering (Lou et al., 2020). Commonly used clustering algorithms are K-means (MacQueen, 1967) and its improved algorithms, fuzzy C-means algorithms, etc. Compared with other clustering methods (Li et al., 2020), K-means clustering algorithm has simple processing algorithms and fast operation speeds for datasets with large data volumes, and the data characteristics between different classes are significantly different, and the clustering effect is better. The K-means clustering algorithm first randomly selects some sets of ultrasound data points, initializes their respective center points, and sets the input sample set D={x1,x2,… xm}, cluster tree k for clustering, maximum number of iterations N. Wheat canopy detection data only need to distinguish between canopy data and non-canopy data, so the number of cluster center points k in the K-means clustering algorithm is set to 2. Suppose the cluster is divided into C={C1,C2,… Ck}, then our goal is to minimize the squared error E, the expression of which is (1):


(1) $$E=\sum_{i=1}^{k}\sum_{x\in C_{i}}║x-{\mu_{i}║}_{2}^{2}$$

where μi is the mean vector of clusters Ci, the center of mass. The expression is (2):


(2) $$\mu_{i}=\frac{1}{\left|C_{i}\right|}\sum_{x\in C_{i}}x$$

The distance from each data point to the centroid is calculated and the data point is classified into whichever class is closest to the centroid. The centroids in each class are used as the new centroids. The above steps are repeated until the center of each class does not change much after each iteration. The center distance of the two classes is compared with the actual height of the spray boom. When the center distance of the two classes is less than 75% of the actual height of the spray boom, it is considered as one class; otherwise, it is considered as two classes.

**Figure 6:**
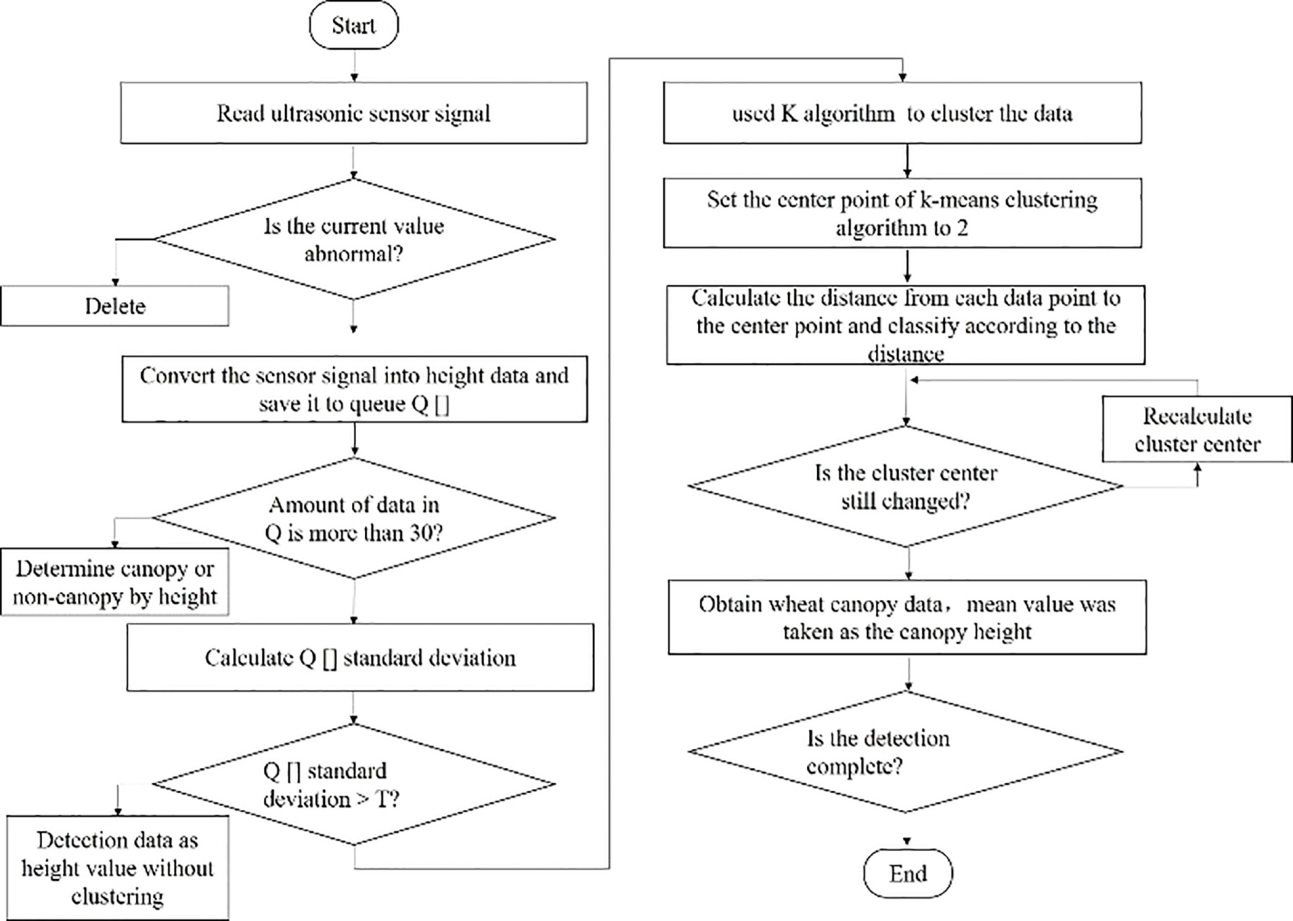
Calculation flow of canopy height based on K-means algorithm. The overall idea of the canopy height calculation process based on K-means algorithm is to first judge whether the detection signal is abnormal data according to the real-time signal of the ultrasonic sensor, and then cluster.

The flow of the canopy height calculation based on the K-means algorithm is shown in **Figure 6**. During operation, the ultrasonic sensor detection system reads the real-time ultrasonic sensor signal and saves it to the first-in-first-out queue Q[]. First, whether the detection signal is abnormal data is judged. If it is abnormal data, the system removes the data from the queue. When the amount of data in Q[] is less than 30, it is directly judged as crown or non-crown based on the set value. When the amount of data in Q[] is greater than 30, the standard deviation σ is obtained for Q[], and if the standard deviation is less than the threshold T, no clustering is required. When the standard deviation is greater than the threshold T, the K-means clustering algorithm is called to pre-process the collected height detection data, determine canopy and non-canopy, and obtain the current canopy height by taking the mean value of the clustered canopy data.

### 2.4 Method of lag distance calculation

**Figure 7:**
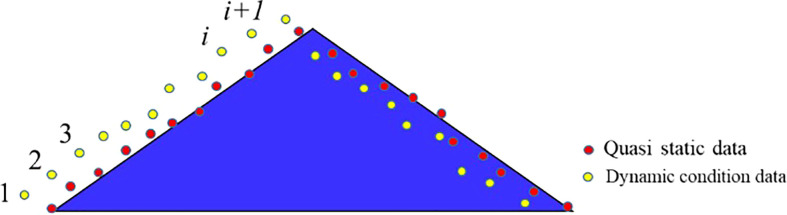
Location diagram of dynamic detection and static detection results. In order to further obtain the relationship between motion speed and ultrasonic detection lag, the detection data obtained at different motion speeds are standard discretized, and the data obtained at no motion speed is compared with 0.05m/s data.

A related study (Zhai et al., 2011) showed that ultrasonic distance measurement sensors have hysteresis. In order to obtain accurate ultrasonic detection hysteresis response characteristics under different speed conditions, the sensor movement speed was set to 0.05m/s, which was considered to be hysteresis-free. The sliding speed of the slider was adjusted to 0.3, 0.6, 0.9 and 1.2 m/s by means of a stepper motor so as to capture the detection distance of the ultrasonic sensor on the step surface under different moving speed conditions. To further obtain a relationship between the velocity of movement and the hysteresis of the ultrasound detection of the target profile, the data points obtained at different velocities were first connected by dash lines and then discrete by sampling intervals for each dash line. From the approximation a more dense profile was obtained and finally the data obtained at no speed of motion was compared with the data at 0.05m/s, as shown in **Figure 7**. The hysteresis amounts were calculated separately.

At a speed of 0.05 m/s, the dense profile data was stored in the array Aq. At dynamic time, the dense profile data was stored in the array Av. The data items of the Ad plants were moved forward by j cells, subtracted from the digits in Aq and then squared as shown in equation (3).


(3) $$C_{\text{j}}=\sum_{i=1}^{N}\left[A_{v}(i+j)-A_{q}(i)\right]$$

where Av is a dense array of shape data obtained when the speed is v; Aq is an array of dense profile data obtained for stationary detection; N is the number of data items stored in each array; Cj is the result of the sum of the differences between Av and Aq after moving forward j lattices. For array Av, the distance of j cells corresponding to the smallest Cj is the hysteresis.

### 2.5 Test design of sensing given-size object

Sound waves propagating in the medium, with the increase in propagation distance, the energy gradually decay. The degree of attenuation and sound wave diffusion, scattering and absorption and other factors are closely related. The sound pressure and sound intensity of the decay law for:


(4) $$\begin{array}{l} P_{x}=P_{0}e^{-\alpha x}\\ I_{x}=I_{0}e^{-\alpha x} \end{array}$$

Where Px, Ix is sound pressure and intensity at x from the sound source;x is the distance between the sound wave and the sound source, m;A is decay coefficient in Np/m (Nepe/m).

**Figure 8:**
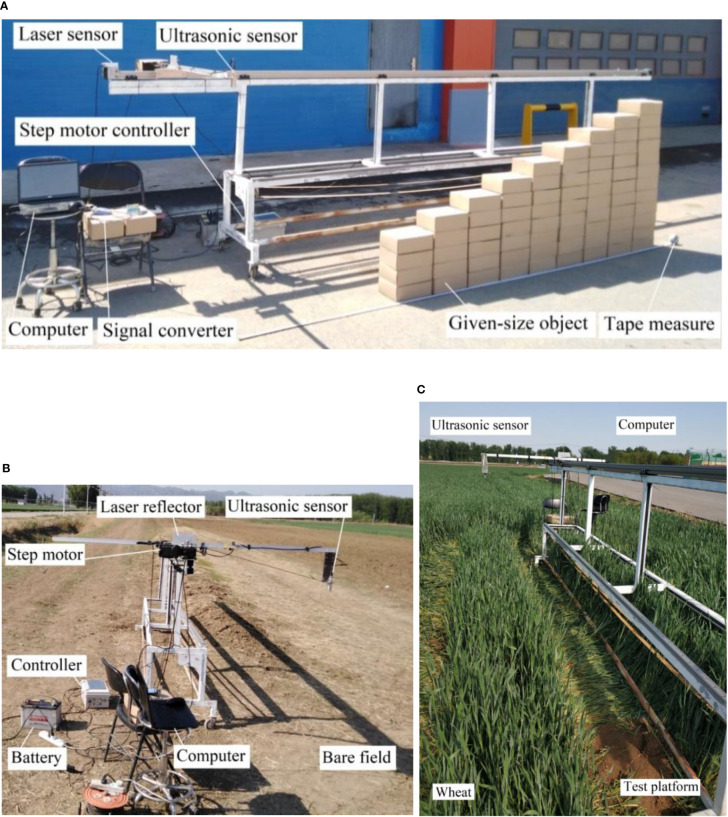
Regular step detection test by ultrasonic sensor. In order to find out the influencing factors and rules of real-time detection by ultrasonic sensors, experiments were conducted under three scenarios, namely, regular steps, bare soil and wheat canopy. **(A)** Regular step detection test by ultrasonic sensor; **(B)** Field ground detection test by ultrasonic sensor; **(C)** Wheat canopy detection test by ultrasonic sensor.

Crop head, local lodging or sudden change of crop canopy height. In order to analyze the relationship between different detection distances and detection values in the moving state, and achieve accurate detection of different detection target heights in the moving process, a regular step detection test was designed, including quasi-static tests and dynamic tests. The test object is an 11-step detection surface consisting of 72 stacked cartons, each measuring 0.30 x 0.20 x 0.10 m. The area of the resulting step surface is larger than the area of a standard plate, and the steps are numbered as shown in **Figure 8A**. The ultrasonic sensor is located directly above the step surface to be measured and the sliding table track is parallel to the step to be measured to ensure that the sensor is always directly above the step when moving with the sliding block. By setting the position of the position limit switch, the travel distance of the sensor to detect the test is limited to 5.0m.

For accurate detection of the height of the sensor from the step surface, the quasi-static test was arranged with reference to (Zhai et al., 2013), where a lower movement speed was selected and the sensor movement speed was set to 0.05 m/s. A tape measure was used to measure the distance of the sensor from the ground and each step surface, and the corresponding measurement data was recorded as the actual distance. The ratio of the absolute value of the difference between the measured data and the detection data to the measured value was taken as the quasi-static height detection error. In the dynamic test, the sliding speed of the sliding block was adjusted to 0.3, 0.6, 0.9 and 1.2 m/s by means of a stepper motor, and the detection distances of the step faces were taken for the going (step up) and returning (step down) ultrasonic sensors at different moving speeds.

### 2.6 Test design of detecting bare field

The detection characteristics of ultrasonic sensors on the ground in the field were investigated for pre- and post-sowing wheat application needs. To simulate a field spray boom detection scenario on undulating ground, bare ground in the field was selected as the detection object so that there were undulations in the terrain during the sensor movement detection. The test setup and set-up was the same as the regular step detection test, divided into static and quasi-static tests, as shown in **Figure 8B**, with a detection ground length of 5.0 m. For the static test, the slider on the push slide table was set to move the sensor forward. A tape measure was applied to measure and record the distance between the ultrasonic sensor and the ground being detected once for every 0.1 m of forward movement of the sensor, with a total of 51 measurement points recorded as the actual distance. The average of the sensor’s detection data within this range for each test was taken as the sensor’s detection distance to the measurement point, using the position of the manual measurement point as the center and a range of 0.1m in front and behind the measurement point. The relative error was calculated from the detection distance and the measurement distance. In the dynamic test, the movement speed of the sensor was adjusted to 0.3, 0.6, 0.9 and 1.2 m/s to collect the detection distance of the ultrasonic sensor on the ground in the field under different movement speed conditions.

### 2.7 Test design of detecting wheat field

In order to investigate the detection characteristics of ultrasonic sensors on wheat canopy, a wheat canopy spray boom height detection experiment was designed using winter wheat before the pulling stage. An area with a length of 5.0m and a width of 4 rows was randomly selected in the direction of the wheat rows. The test platform was positioned so that the area to be measured was parallel to the crop rows and the ultrasonic sensor was positioned directly above the selected area, as shown in **Figure 8C**.

**Figure 9:**
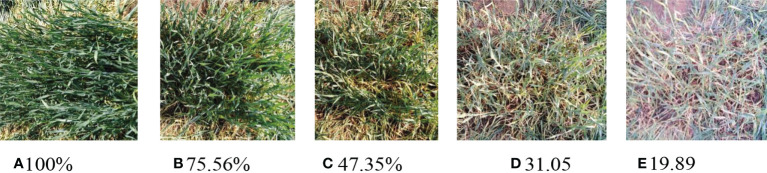
Field ground detection test by ultrasonic sensor. The canopy with different density was formed by randomly and evenly cutting the original wheat four times. **(A)** 100% **(B)**75.56% **(C)** 47.35% **(D)** 31.05 **(E)** 19.89.

Once the test platform was in place, the slider on the sliding table was manually pushed to move the sensor forward. A tape measure was employed to measure and record the distance between the ultrasonic sensor and the wheat canopy plane at the corresponding position for every 0.1m of forward movement of the sensor. During the quasi-static test, in order to achieve the comparison of detection results of wheat with different canopy densities, this paper conducts manual random pruning of the original growth wheat, with a height of 5cm above the ground, randomly and evenly prune some wheat plants, weigh and record the quality of the pruned wheat for a total of four times. Different wheat canopy densities were obtained by different levels of pruning. The wheat canopy density was expressed as the percentage of the wheat weight after pruning to the original weight. The original growth density of wheat was defined as 100%. Set the target of 4 cuts to be about 25%, 20%, 15% and 10% of the current density as show in **Figure 9**. The amount of 4 times of cutting and the weight of the remaining weight after cutting are shown in **Table 1**.

**Table 1:** Statistics of four pruning of wheat canopy.

	Cut off times				residual weight	total weight
	1	2	3	4		
Cut off weight	1489.6	1719.9	993.8	680.5	1212.1	6095.9

The sensor movement speed was also set to 0.3, 0.6, 0.9 and 1.2 m/s for the wheat canopy detection trials at different canopy densities, with three replications of each trial.

## 3 Results

### 3.1 Ultrasonic sensor calibration

**Figure 10:**
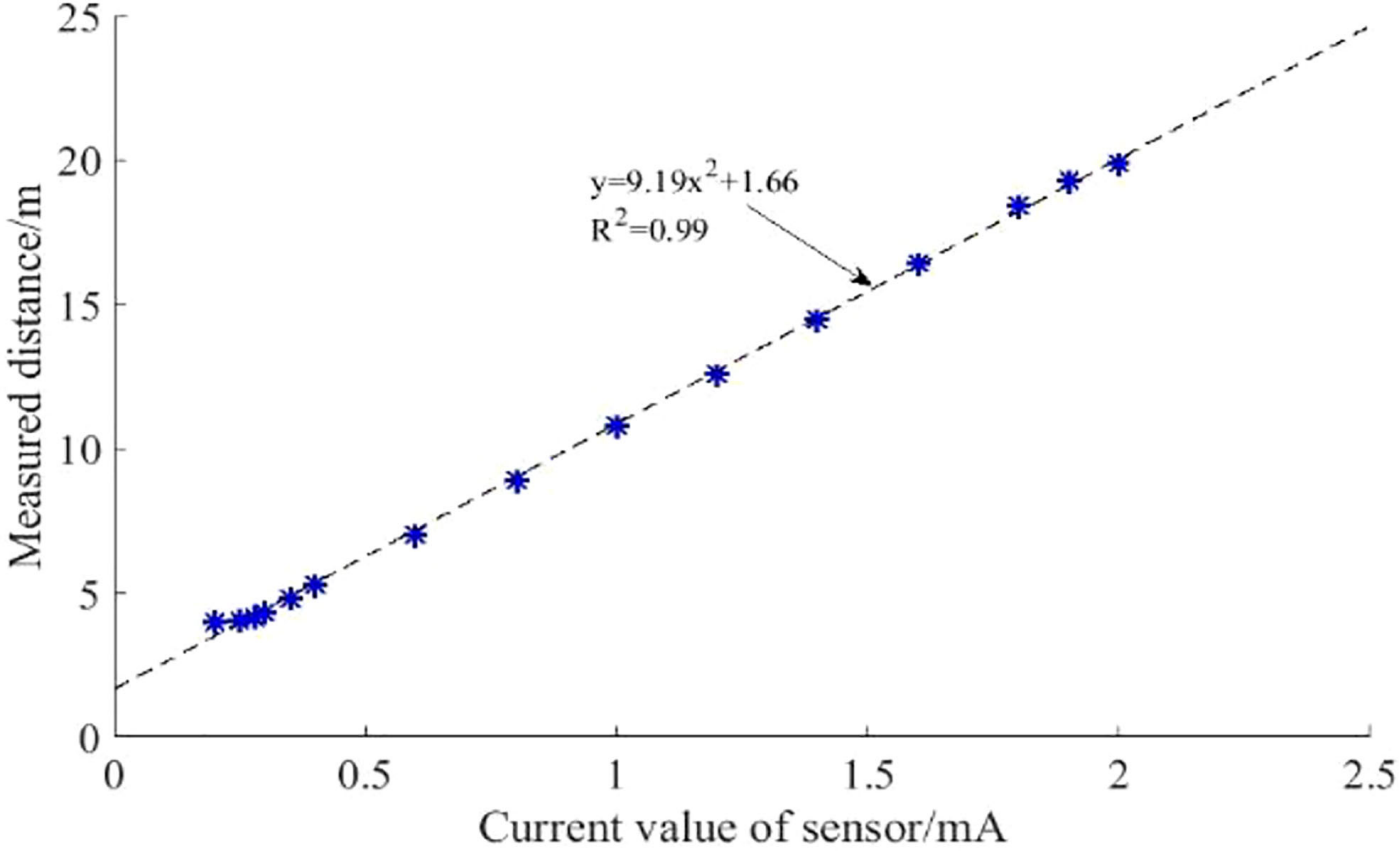
Wheat canopy detection test by ultrasonic sensor. The sensor current increases with the increase of measuring distance, with good linear correlation, and the correlation coefficient reaches 0.99. Symbol “*” represent distance detection values corresponding to different voltages.

The relationship between the ultrasonic sensor current and the measurement distance is shown in **Figure 10**, where the sensor current increases with increasing measurement distance. The sensor output currents at detection distances of 0.2m and 2.0 were 4mA and 19.90mA respectively. A linear fit to the detection data gives the mathematical equation y=9.19x2+1.66, where R2 = 0.999, it shows that there is a high linear relationship between variables x and y, and the linear correlation is large and the fitting accuracy is high. However, at detection distances of 0.2-0.3m, the linearity of the sensor output signal values is relatively poor. If the sensor detects the height of the spray boom in this detection range, large detection errors may be generated. When used, this detection range shall be avoided.

**Figure 11:**
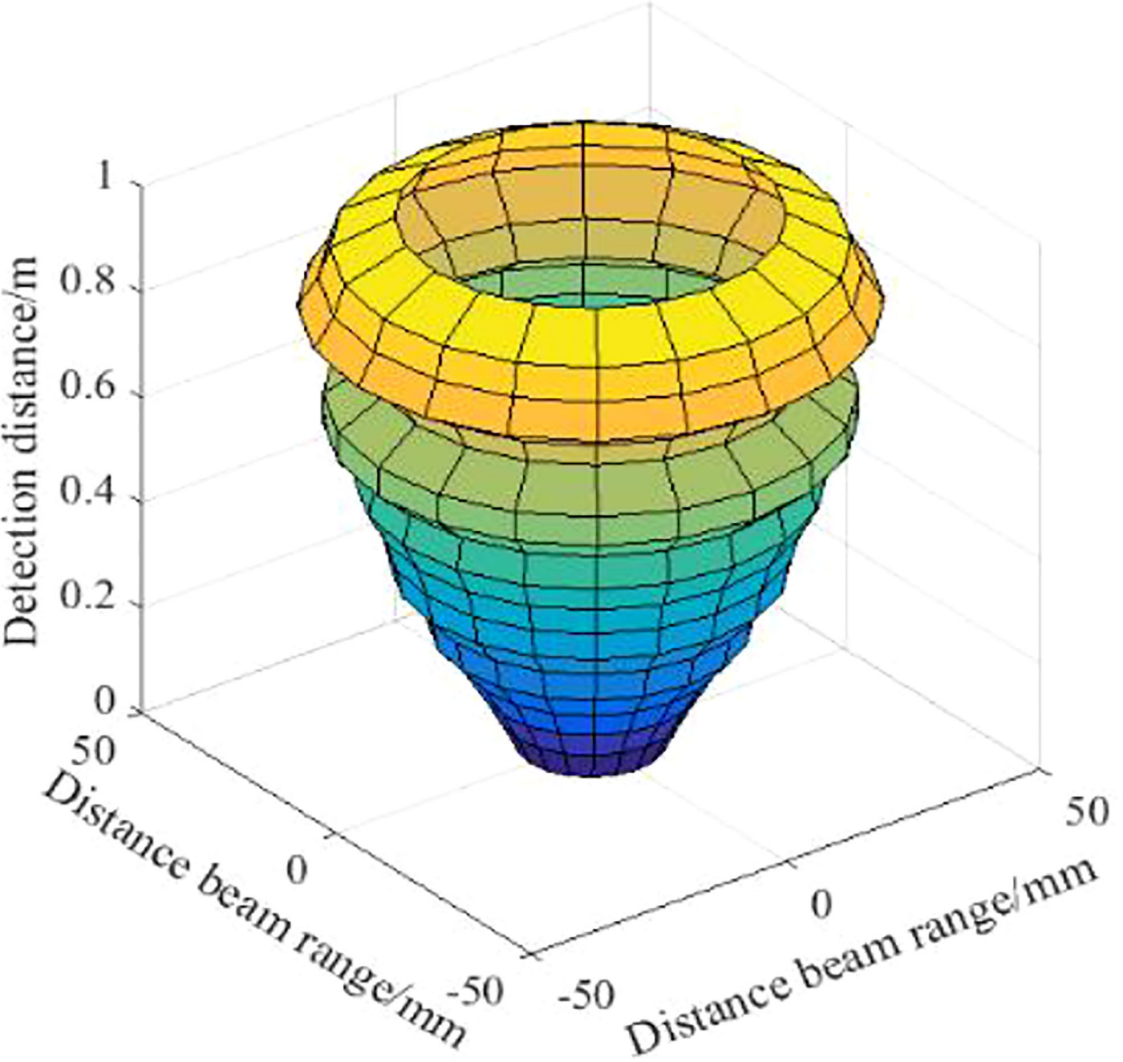
Wheat with different canopy density. The range of the acoustic cone of the ultrasonic sensor is “spindle”, and the width of the acoustic cone increases linearly with the increase of the detection distance.

The range of the ultrasonic transducer’s sound cone is shown in **Figure 11**, the sound cone is “spindle” shaped. In the range of 0.3-1.3m, the cone width increases linearly with the detection distance as a whole. At a detection distance of 1.3-1.7m, the cone width varies more dramatically. At a detection distance of 1.6m, the cone width is at its maximum, with a maximum cone width of 45.8cm and a maximum effective detection distance of 2.0m.

### 3.2 Test result of detecting given-size object

#### 3.2.1 Detection results under quasi-static conditions

The sounding height data obtained at a sounding speed of 0.05m/s are shown in the **Table 2**.

**Table 2:** Detection height data at.05m/s velocity.

Step No.	Detection value		Actual value	Mean value of error/%
	Outbound	Inbound		
1	1.049	1.047	1.027	2.1
2	0.924	0.921	0.908	1.7
3	0.813	0.816	0.802	1.7
4	0.702	0.705	0.694	1.4
5	0.601	0.600	0.593	1.3
6	0.492	0.492	0.487	1.1
7	0.376	0.379	0.374	1.0
8	0.273	0.272	0.271	0.7
9	0.162	0.163	0.161	1.2
Average				1.35

**Figure 12:**
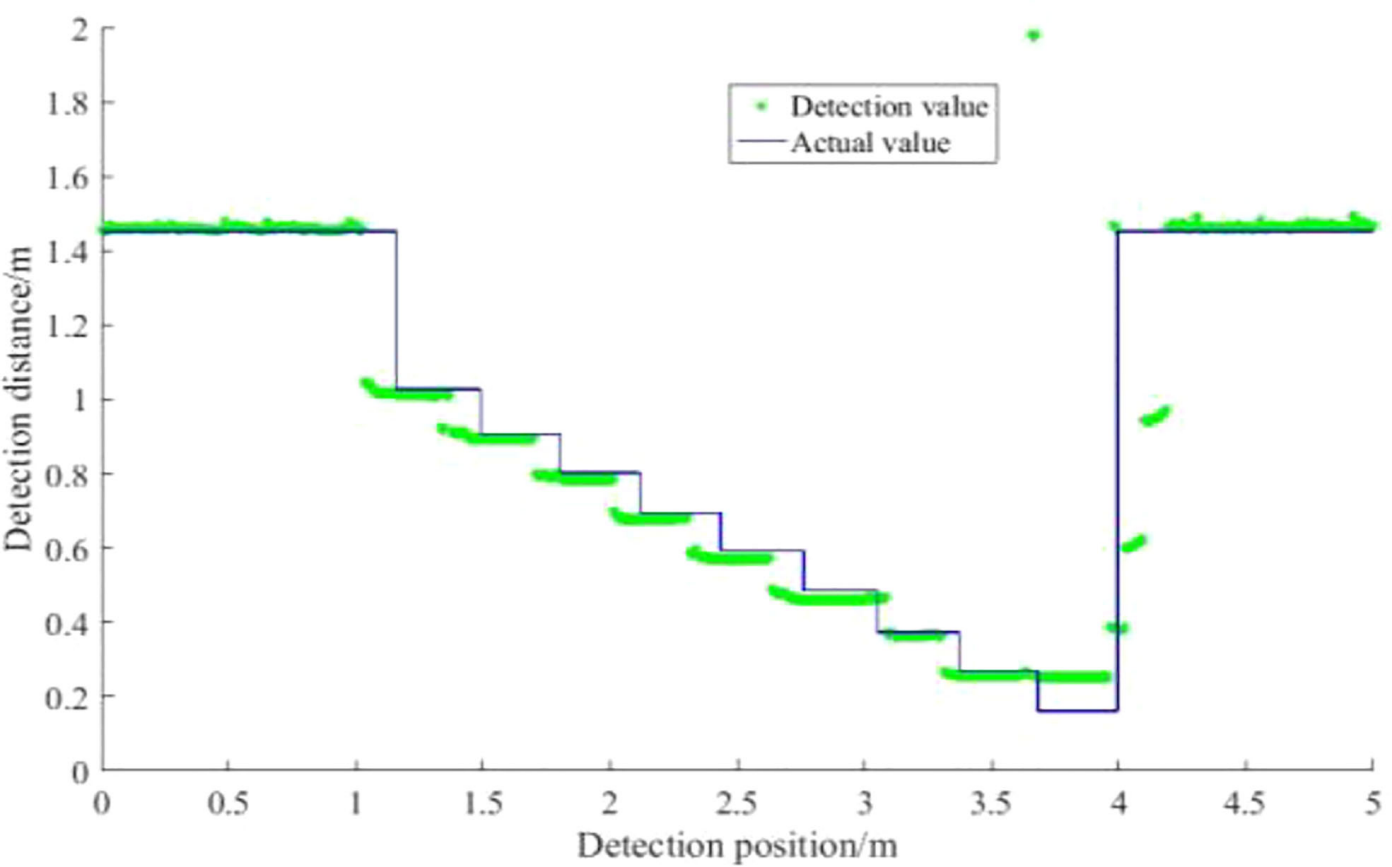
Calibration curve of ultrasonic sensor. The comparison between the detection value of the ultrasonic sensor and the actual value in the quasi-static condition shows that the detection has high accuracy.

The forward high detection error is 1.3%, the backhaul detection error is 1.4%, and the average detection error is 1.35%. It shows that the ultrasonic sensor has high accuracy in detecting the distance of the step surface under quasi-static conditions. The pairing of the probe value with the actual value is shown in **Figure 12**.

The detection of the sensor at the change of the step surface is ahead of the actual position of the step, mainly attributed to the fact that the ultrasonic sensor has a certain width of the acoustic cone. In the direction of movement of the sensor, the height of the step increases in steps (the distance of the sensor from the step surface decreases in steps). Because of the width of the sensor beam, higher step surfaces can still be detected when the sensor is at a lower step position. This phenomenon, although the ultrasonic sensor detection results in position error (in the actual distance of the larger feedback distance signal), but in the spray boom height detection in time to effectively detect the spray boom closer to the obstacle. The spray boom control system should raise the height of the spray boom in time to avoid a collision with the spray boom.

**Figure 13:**
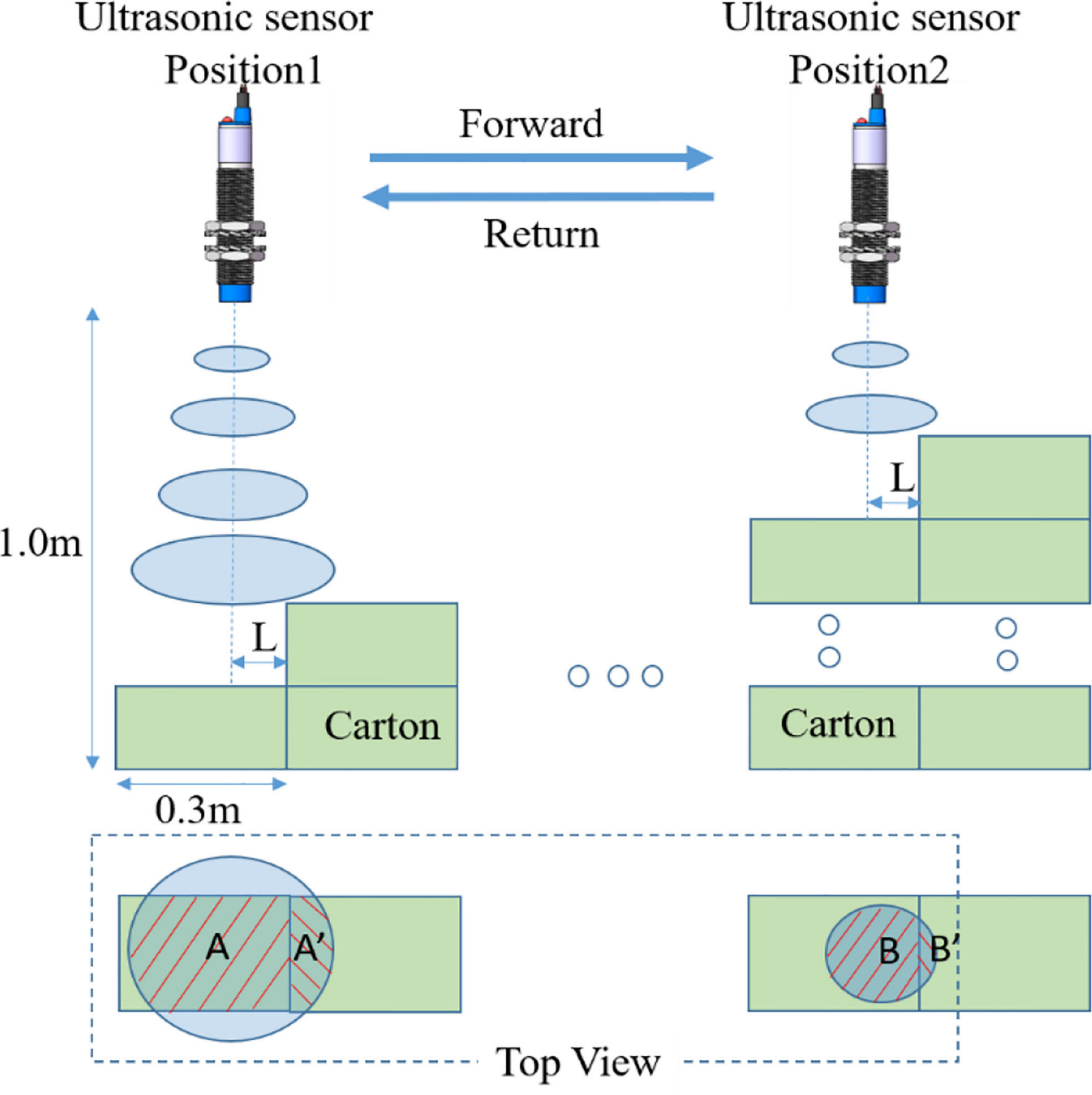
Ultrasonic cone. The quasi-static test ignores the influence of velocity and the detection area changes due to the change of detection distance, and the detection area affects the measured value at the step transformation.

The quasi-static test allows the effect of velocity to be ignored, so it is considered to be due to the change in detection area caused by the change in detection distance, with different amounts of overtopping caused by the detection area. As the distance of the ultrasound transducer from the step face gradually decreases during the go-round, the detection area formed by the ultrasound transducer at the step face gradually decreases, as can be seen from the obtained acoustic cone range of the ultrasound transducer. By the 9th step, a detection blind zone appears due to the close distance between the transducer and the step surface, exhibiting an overlap of detection distances at the 8th and 9th steps. As depicted in **Figure 13**, the detection area is greater at position 1 than at position 2. When the sensor is in the step position, the smaller the detection area the smaller the amount of overrun caused. Detection position 1 forms an area A’ at the step surface that is larger than the detection area B’ formed at the step surface at detection position 2. Therefore, at larger detection distances, the more pronounced the vibration or jitter of the sensor due to movement, the more unstable the detection area and the greater the fluctuation of the detection data.

As can be seen from the **Table 2**, with the increase in detection distance, the detection radius of the ultrasonic sensor gradually increases, the detection distance at the step to produce a switch in the area of B is gradually increased, When the detection distance is switched, the actual offset between the sensor and the step gradually increases and then tends to be stable, with an offset of 10.4cm. It can be concluded that when the detection distance is 0.271-1.027m, the smaller the detection distance, the smaller the detection range, the concentration of acoustic energy, B area smaller than the target plate area can produce sufficient reflected wave energy.

When the detection distance gradually increases, a larger B area is required before the reflected wave energy reaches the detection distance switching threshold, so the B/A keeps increasing when the detection distance is switched.

#### 3.2.2 Detection results under different speed conditions

**Figure 14:**
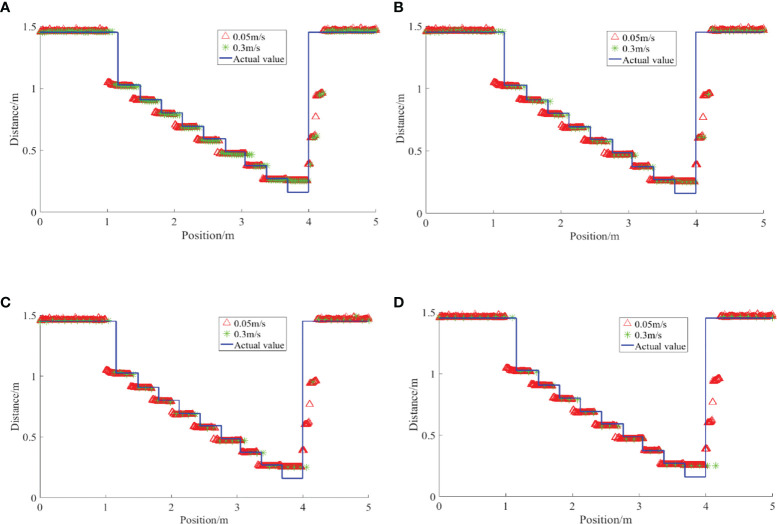
Detection results of regular steps by ultrasonic sensor under quasi-static conditions. The number of sampling points is different at different speeds, and the fluctuation of detection offset increases gradually with the increase of speed **(A)** 0.3 m/s **(B) **0.6m/s **(C)** 0.9m/s **(D)** 1.2m/s.

The detection results of the ultrasonic sensor on the step surface under different moving speed conditions are shown in **Figure 14**.

The detection results in motion conditions are less offset relative to the actual position of the step than in quasi-static conditions. On the one hand, the ultrasonic sensor receives the return wave during the emission cycle during motion, and the distance generated by the motion causes a certain hysteresis. However, due to the high ultrasonic wave transmission speed and the relatively short detection distance, the amount of position offset caused is negligible. On the other hand, the position offset is related to the acquisition frequency of the ultrasonic sensor, the shorter the acquisition period the smaller the hysteresis. As shown in **Figure 14C**, the number of acquisitions at a speed of 0.03m/s is significantly higher than at 1.2m/s. The sampling frequency of the sensor is 10Hz, and the sampling time point and the relative position of the step also affect the actual hysteresis. However, as the speed increases, the offset relative to the quasi-static condition becomes greater.

**Figure 15:**
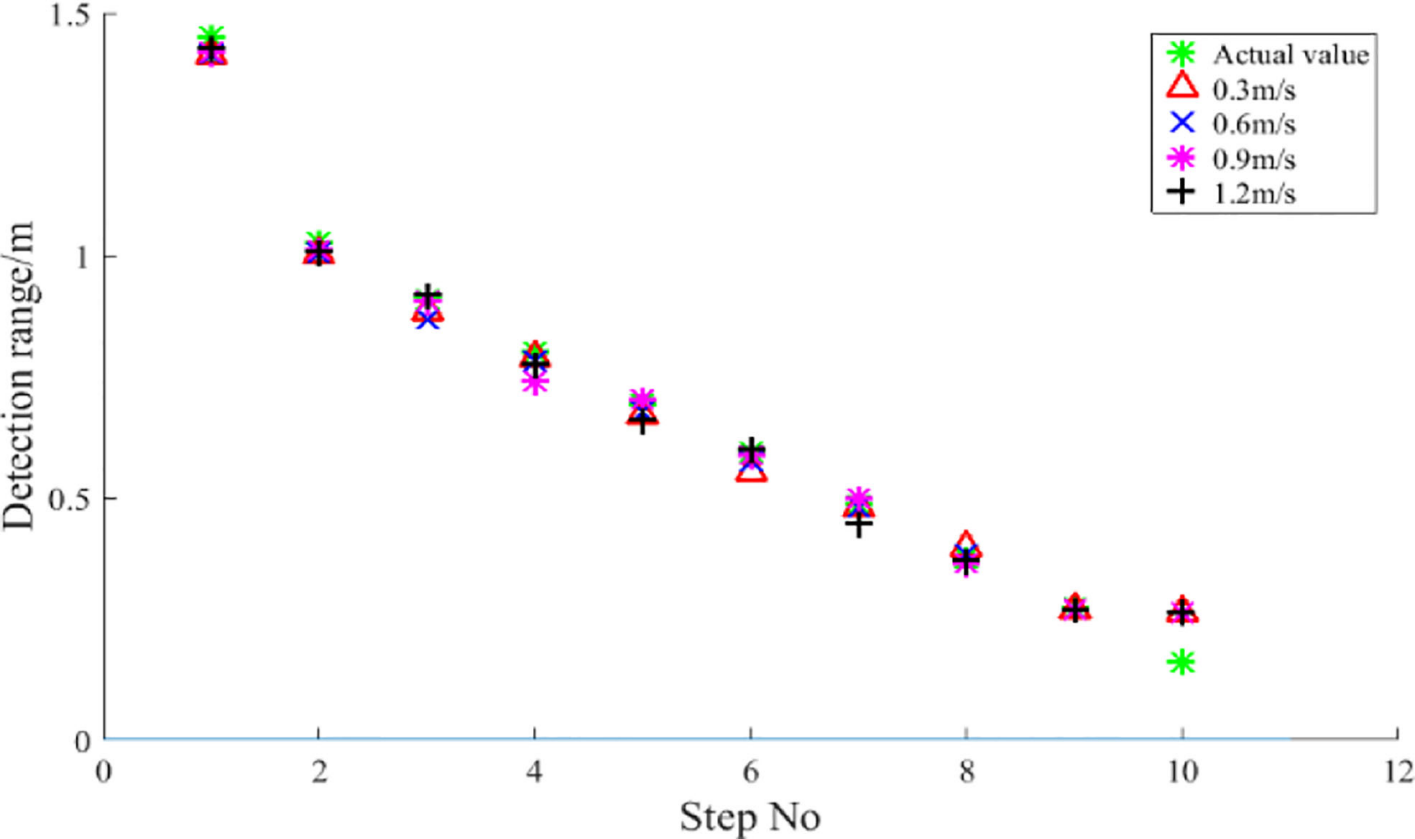
Detection range of step surfaces with different heights. The average value of the detection data at different speeds within the range of the step is the statistical result of the detection distance.

The average of the detection data (with the burr signal removed) over the range of the step at different speeds is taken as the sensor’s detection distance to the step and the statistics are shown in **Figure 15**.

As can be seen from the figure, the detection values at different speeds are larger than the measured values at the 10th step. The relative error rates of the detection distances at 0.05, 0.3, 0.6, 0.9 and 1.2 m/s are 63.54%, 56.21%, 56.33%, 56.46% and 56.27% respectively, which is a large detection error. The main reason for this is attributed to the fact that the ultrasonic sensors have a certain detection blind spot. The sensor used in the study has a detection blind spot of 0.20 m. As can be found in **Table 3**, the actual distance of the ultrasonic sensor from the step surface at the 10th step is 0.161 m, which is already in the sensor’s detection blind spot. In order to eliminate the large errors in this condition, it is necessary to study the characteristics of spray boom height variation in the field based on the ultrasonic sensor for spray boom height detection. The range of spray boom height variation in the field needs to be estimated and the sensor installed at a suitable position on the spray boom so that the detection distance of the spray boom height is always within the effective detection range of the sensor. The results at steps 1-9 indicate that the detection distance is not significantly affected by speed in the range 0.3-1.2 m/s, with a mean detection distance deviation of 4.1 cm. The sensor has a relatively large detection error at 1.2 m/s for larger detection distances. In particular, at the 5th step, the relative error rate of the detection distance is as high as 15.48%. The detection error is mainly due to the hysteresis of the ultrasonic sensor signal feedback (Zhai et al., 2011).

**Table 3:** Switching of detection values at steps for different detection heights.

Distance/m	Detection radius/cm	A_ds_/cm^2^	B_ds_/cm^2^	B_ds_/A_ds_ (cm^2^)	O_ds_/cm
0.271	9	180.86	20.09	0.1	8
0.374	10.7	265.89	29.54	0.1	9.5
0.487	11	279.46	34.54	0.11	9.7
0.593	11.8	322.29	43.94	0.12	9.9
0.694	12.3	348.82	52.12	0.13	10.1
0.802	12.9	386.85	57.80	0.13	10.3
0.908	13.6	423.73	74.77	0.15	10.4
1.027	14.1	539.98	75.59	0.14	10.4

A_ds_, Area A when detection distances switching.

B_ds_, Area B when detection distances switching.

O_ds_, Actual offset of the sensor from the step when the detection distance is switched.

#### 3.2.3 Detect process anomaly data

As can be seen from **Figure 14**, the ultrasonic sensor detects the step surface with erroneous data (glitch signal). The percentage of the total detection data by counting the number of glitch signals that occur at each movement speed of the sensor, the signal glitch rate when detecting the regular step surface as an ultrasonic sensor, as shown in the **Table 4** (the total data of the 3 repeat tests is counted in the table). The signal glitch rate of the ultrasonic sensor when detecting regular steps is up to 0.22%. The incidence of this signal burr is low in the spray rod height detection and is acceptable.

**Table 4:** Signal glitch rate of ultrasonic sensors for detecting regular step.

Speed m/s	Total number of probe data, pcs	Number of burr signals, pcs	Average signal burr rate, %
0.05	2762	6	0.22
0.3	469	0	0
0.6	240	0	0
0.9	163	0	0
1.2	127	0	0

### 3.3 Test result of detecting bare field

**Figure 16:**
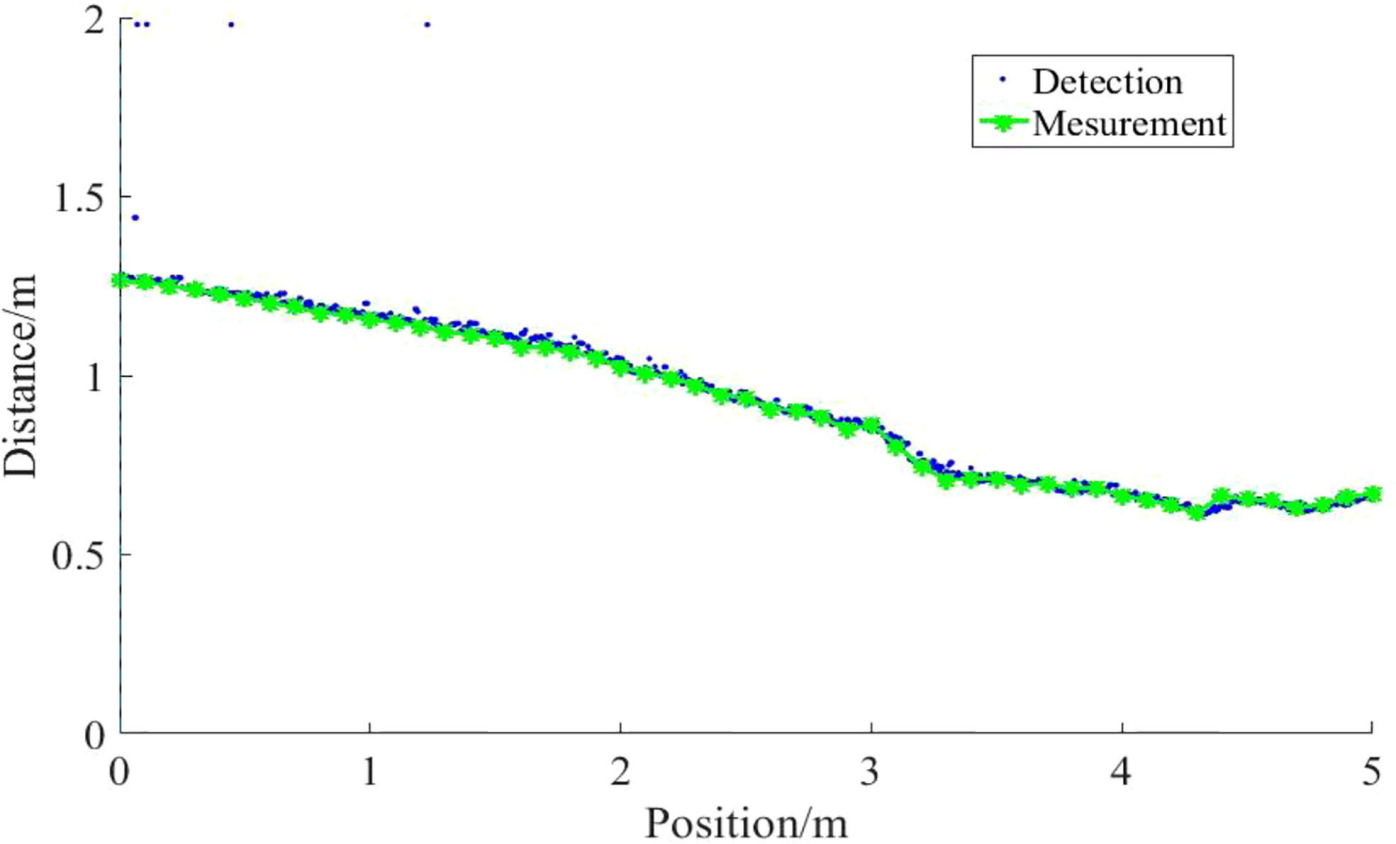
Detection distance of ultrasonic sensors on regular step at different speeds. The height detection results of the field ground under manual measurement and quasi-static conditions show that, due to the flat terrain, the detection results of the ultrasonic sensor on the field ground do not lead or lag behind the actual position.

The results of the height detection of the field surface under manual and quasi-static conditions are shown in **Figure 16**. By counting and averaging the errors at each measurement point, the relative error between the detection distance and the measurement distance was obtained as 1.4%. The ultrasound sensor did not show any over-estimation of the detection results relative to the actual position of the field surface, mainly because the terrain of the target field surface changes gently, which significantly reduces the influence of the ultrasound sensor beam width on the detection results.

**Figure 17:**
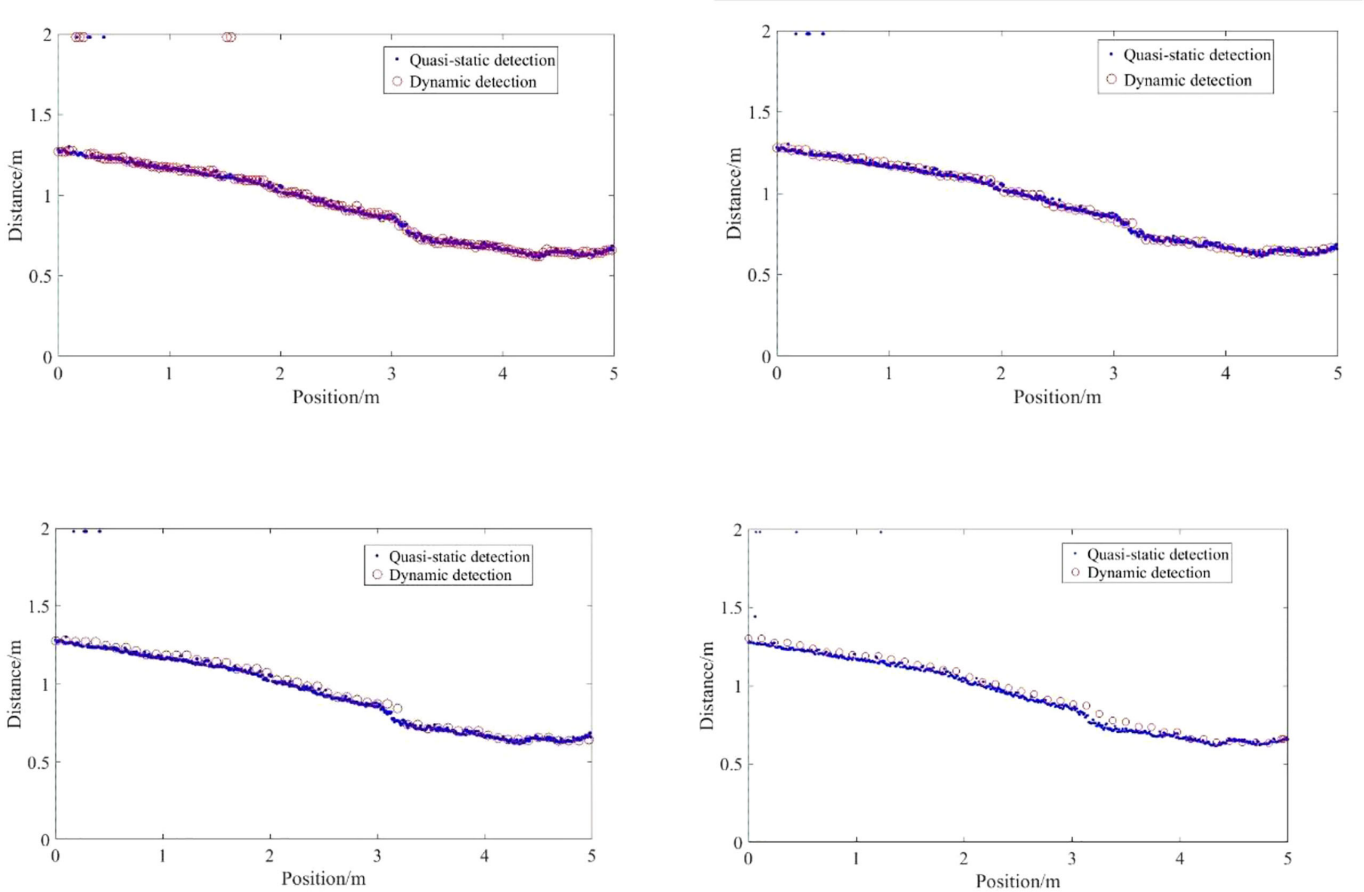
Detection distance of ultrasonic sensor for the regular steps. During the detection of bare soil, there is no obvious lag of the relative quasi-static actual position 0.3m/s, 06 m/s, 0.9 m/s, 1.2 m/s.

The results of the ultrasonic sensor’s detection distance to the field surface at four speeds of 0.3, 0.6, 0.9 and 1.2 m/s for different moving speed conditions are shown in **Figure 17**. The detection distance of the ultrasonic sensor on the field surface is more accurate and there is no significant lag in the detection results relative to the actual quasi-static position in the regular step detection test at the four operating speeds. It is mainly due to the gentle terrain changes in the ground. The ground surface in the field generally increased gradually along the direction of sensor movement (the distance of the sensor from the ground decreased step by step), while the ground surface changed gently and there were almost no dramatically changing ground. However, when comparing the detection distance between the two speeds of 0.3m/s and 1.2m/s, the detection distance error of 1.2m/s is relatively large. The detection distance error is mainly caused by the detection lag, which is the distance travelled in the interval between samples. Therefore, the lagging distance gradually increases with the increase of speed.

**Figure 18:**
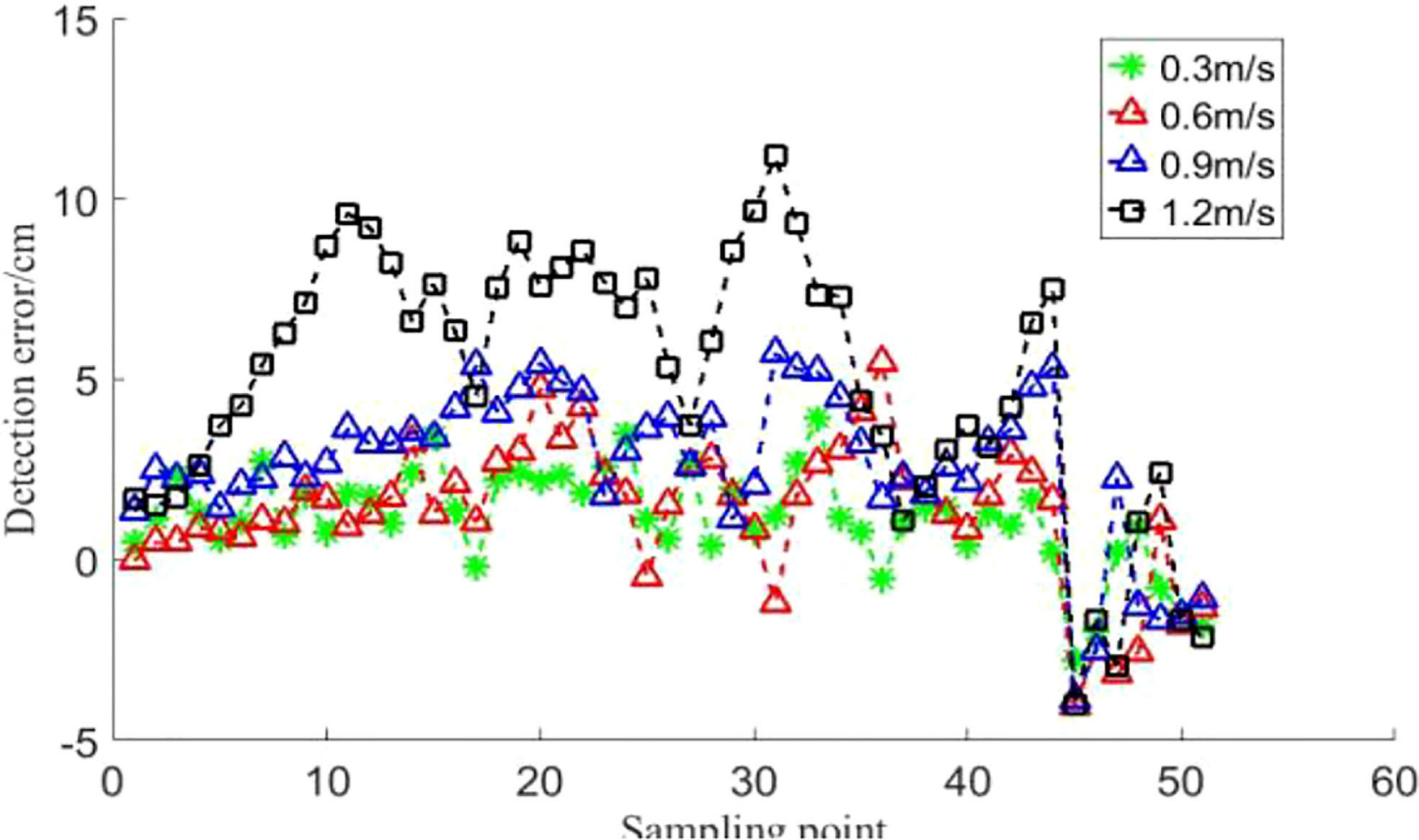
Detection results of ultrasonic sensors on field ground. When the moving speed of the ultrasonic sensor is lower than 0.9 m/s, the average relative error of the detection distance is lower than 5%, and when the moving speed is 1.2 m/s, the maximum relative error is up to 14.79%.

The detection distance of the ultrasonic sensor nearest to the 51 measurement points was counted, and the difference between this detection distance and the measured distance of the 51 measurement points was taken as the relative error, and the statistical results are shown in **Figure 18**. The average value of the relative error of the detection distance is less than 5% when the ultrasonic sensor is moving at a speed not exceeding 0.9 m/s. At a moving speed of 1.2 m/s, the relative error of the ultrasonic sensor detection distance is large, with a maximum relative error of 14.79%. The relative error at 30 sampling points is significant and the phenomenon is mainly caused by the relatively large ground variation.

It can be observed that slope and velocity together affect the accuracy of the detection distance during the detection tests on bare ground. When the overall slope of the soil slope is low, the advance distance is not increased as the detection range increases. Comparative step experiments show that the advance distance is mainly influenced by the slope. When the slope is low, the detection range has no effect on the advance distance and the terrain on the field surface changes gently. Therefore, the detection overrun or lag due to the detection range can be ignored.

Under sloping ground slope conditions, the effect of velocity on the ultrasonic sensor is not significant and the detection distance error is mainly caused by the lagging distance. When the speed is 1.2 m/s, the mean value of the relative detection distance error increases sharply. It follows that the sprayer should move at a speed of less than 1.2 m/s when performing ultrasonic sensor-based spray boom height detection.

Burr data also existed for the detection of the ground in the field by the ultrasonic sensor, and the burr rate of the detection signal at different movement speeds of the sensor is shown in **Table 5** (the statistics in the table are for the total data from three replicate trials). The maximum burr signal incidence was 2.15%. This signal burr incidence is also low in the spray boom height detection due to the large total number of detection signals, which can be removed by filtering in the signal processing.

**Table 5:** Signal glitch rate of ultrasonic sensors on field ground.

Speed m/s	Total number of probe data, pcs	Number of burr signals, pcs	Average signal burr rate, %
0.05	2760	24	0.87
0.3	464	10	2.15
0.6	238	0	0
0.9	162	3	1.85
1.2	127	0	0

### 3.4 Test result of detecting wheat field

#### 3.4.1 Quasi-static wheat height detection results

**Figure 19:**
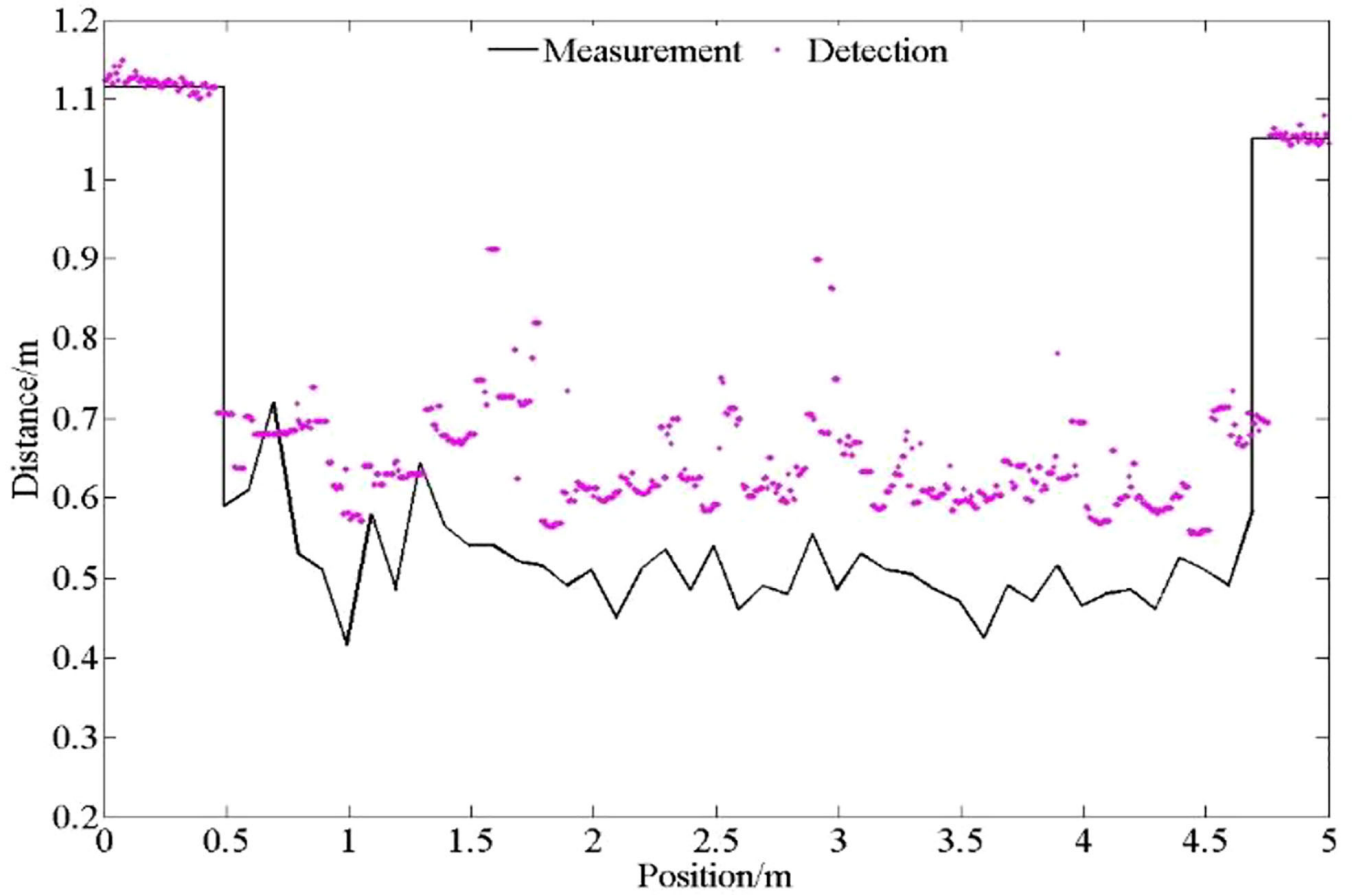
Detection distance of ultrasonic sensors on field ground at different speeds. The detection data of wheat canopy by ultrasonic sensor are discrete.

The results of the wheat canopy detection under quasi-static conditions are shown in **Figure 19**.

**Figure 20:**
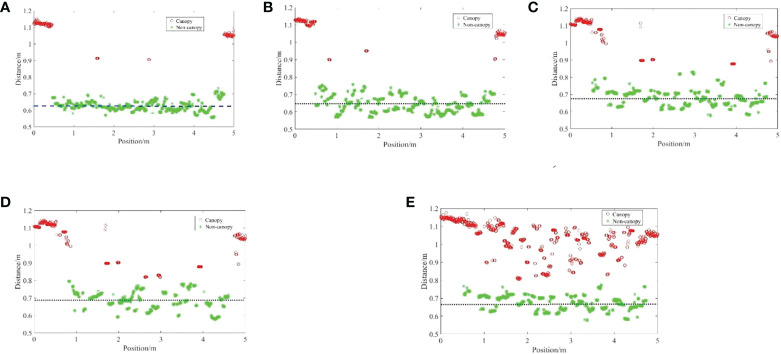
Detection distance relative error of ultrasonic sensor on field ground. The K-means clustering algorithm is used to cluster the data under five density conditions, realizing the segmentation of canopy and non canopy detection data. **(A)** Distance detected by the ultrasonic sensor to the 100% wheat canopy; **(B)** Distance detected by the ultrasonic sensor to the 75.56% wheat canopy; **(C)** Distance detected by the ultrasonic sensor to the 47.35% wheat canopy; **(D)** Distance detected by the ultrasonic sensor to the 31.05% wheat canopy; **(E)** Distance detected by the ultrasonic sensor to the 19.89% wheat canopy.

As can be seen from **Figure 20**, the ultrasonic sensor can distinguish the area before and after the wheat band, but the detection data of the wheat canopy is more discrete, because the detection object of the wheat belt is the sparse and uneven wheat canopy. Part of the probe data is the feedback of the ultrasonic beam to the wheat canopy, while the other part of the data may be the feedback of the beam to the non-canopy object of the wheat gap.

**Figure 21:**
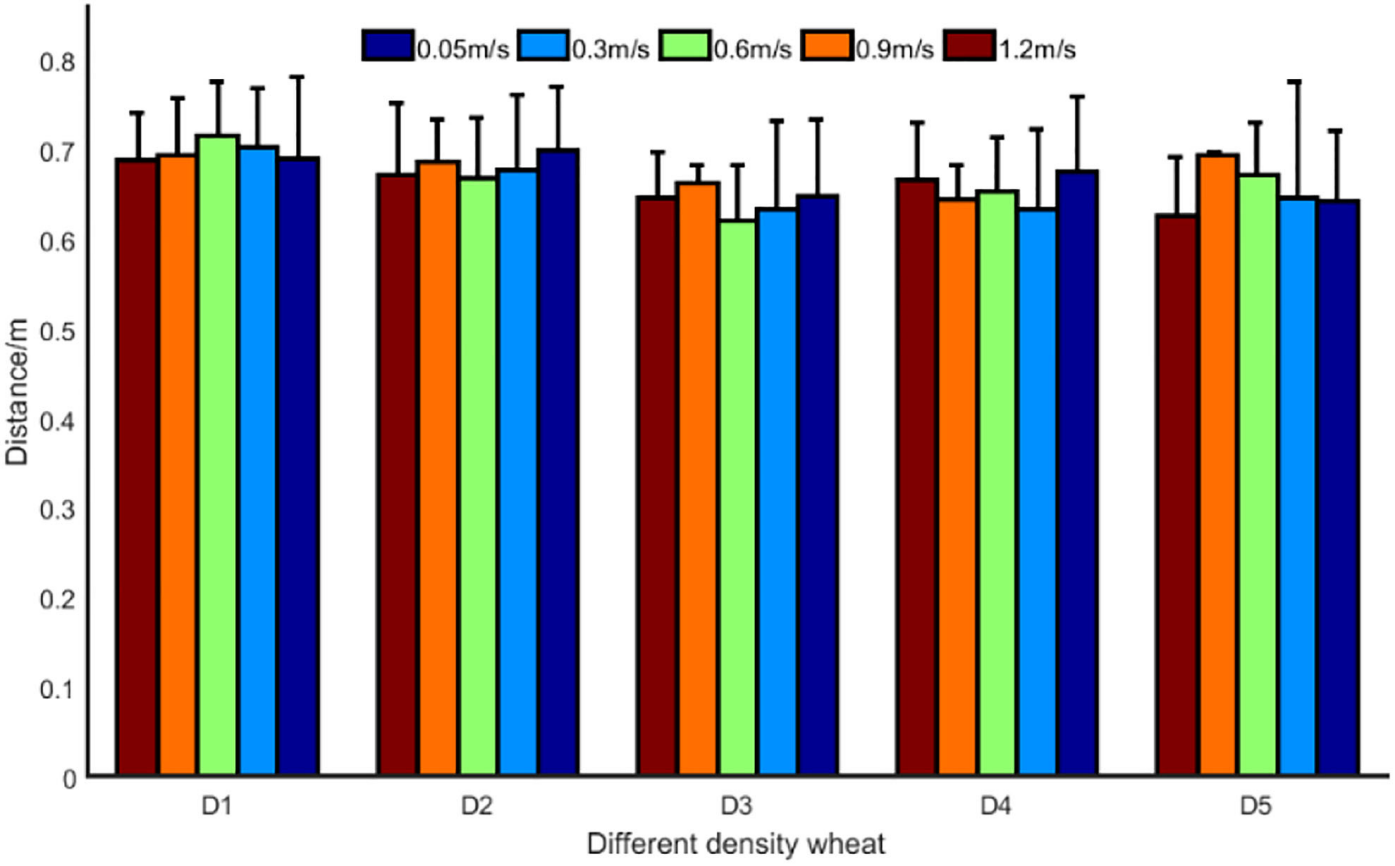
Comparison of wheat canopy and non-canopy with different density. The detection speed has no obvious influence on the detection value.

The K-means clustering algorithm is used to cluster the data under five density conditions, as shown in **Figure 21**. Through the clustering algorithm, the segmentation of canopy and non-canopy detection data is realized, and the wheat canopy data is obtained.

**Figure 22:**
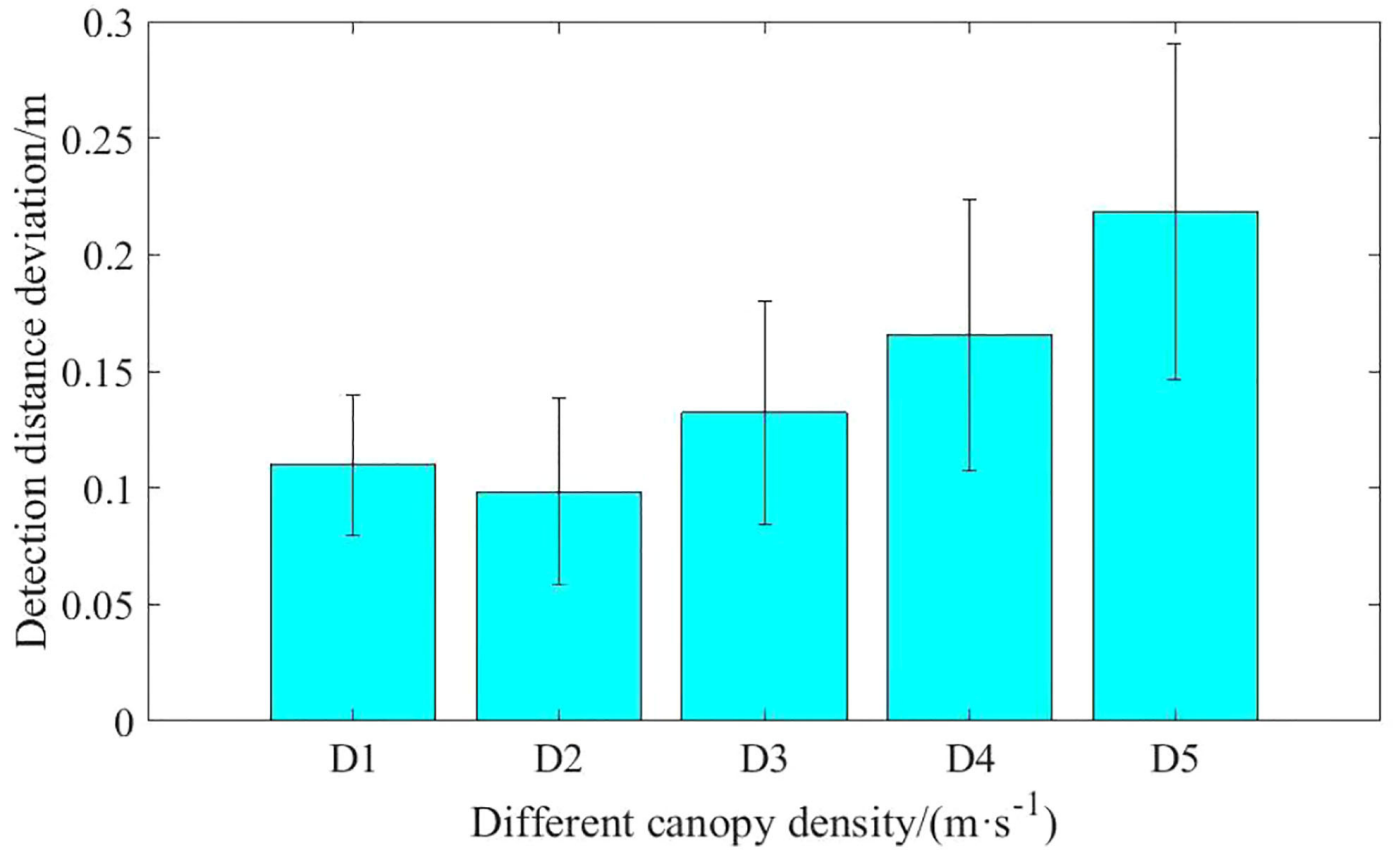
Detection results of ultrasonic sensors on wheat canopy. The thinner the canopy, the greater the detection error.

The mean value of the manual measurement for wheat was 0.52 m. Overall, the detection distance was large relative to the manual measurement distance. The results of the ultrasonic sensors on the wheat canopy are influenced by the density of the wheat and the detection bias tends to increase as the density of the wheat decreases. Because the wheat canopy does not have a regular beam reflection plane, the ultrasound beam can detect the plant stalks and branches below the canopy, and even the pulses are more likely to miss the crop and reflect back from the ground during wheat canopy detection. In addition, the spatial characteristics of plant leaves are related to their height and growth stage. As the wheat was in the pre-gestation stage, the leaves of the wheat were still in the growth process and the leaves were mostly slope shaped, with very few being parabolic. The results for different canopy densities are shown in **Figure 22**.

The sparser the canopy, the greater the detection error (up to 0.22m), and the error tends to increase (up to 0.07cm2). The smaller the wheat density, the more obvious the dispersion of detection results. When the density of wheat is small, the ultrasonic sensor detects the wheat leaves, but it is difficult to form a relatively stable echo, the received echoes may be reflected between the leaves and stems, or reflected back to the sensor from multiple leaf surfaces, resulting in the detection of wheat height fluctuations (Aziz et al., 2004). The lower the wheat density, the more the sensor detects non-canopy results, which are mainly wheat leaves near the ground or at ground level, resulting in greater fluctuations in height information.

#### 3.4.2 Detection height results of wheat under moving conditions

According to the clustering algorithm given above, the detection data obtained by each experiment is extracted to extract the feedback data of the ultrasonic sensor beam to the wheat canopy. The average of the distance detected by the ultrasonic sensor to the wheat canopy is then calculated, as shown in the **Figure 21**.

From the detection distance data for the same density of wheat, it can be seen that there is no correlation between detection error and velocity. However, the detection errors for velocities of 0.3m/s-1.2m/s were all smaller than the detection errors in quasi-static conditions. It indicates that the average height of the reflective surface of the wheat canopy increases when the sensor detects the canopy at a certain speed. In other words, the continuous measurement method reduces the interference caused by irregular reflective surfaces under static conditions. The continuous measurement method outperformed the quasi-static measurement method and achieved better results (Pittman et al., 2015). The mean value of the detection results varied little between different movement speeds, with the maximum difference being 0.062 m at Density5 and the minimum difference being 0.022 m at Density1, i.e. there was no significant effect of detection speed on the detection values.

Generally, the sensor showed an increasing trend for each movement speed detection with decreasing wheat density. It indicates that the smaller the canopy density, the less ultrasonic waves are reflected from the canopy and the more ultrasonic waves are reflected by the stems and leaves below the canopy. When moving at a speed of 0.3m/s-1.2m/s, the range of deviation of the ultrasonic sensors for D1, D2, D3, D4 and D5 wheat canopies was 0.10-0.12 m, 0.09-0.12 m, 0.11-0.16 m, 0.13-0.18 m and 0.19-0.20 m, respectively. The average detection deviation was 0.14 m. The results were compared with the work of Kataoka et al. (2002), who used ultrasonic sensors to measure the height of soybeans and maize, achieving accuracies of 0.03 and 0.10 m, respectively. It was concluded by the authors that as soybean is a broadleaf crop the leaf surface is horizontal, i.e. perpendicular to the ultrasonic sensor, whereas in maize the leaves grow at an angle to the vertical. Similar to maize wheat provides a smaller target for the ultrasonic pulses and a relatively larger detection bias.

To obtain more accurate canopy distances, wheat canopy detection bias needs to be corrected appropriately, and different height percentiles can also be used. According to a previous study by Scotford and Miller, the 90% percentile of each data set provided the best estimate of wheat plant height (Scotford and Miller, 2004), but the size of the percentile was related to the leaf area, leaf angle, number of non-leaf parts, etc. of the crop (Shibayama et al., 1985). Bronson suggested using the 75% ultrasound measured plant height data as it provides a more accurate estimate of cotton height than the mean or median (Bronson et al., 2020). For the wheat growing period in this study, the percentile size used was determined as **Table 6**, based on different densities of sounding height versus manual measurement data and a field ultrasound to ground distance of 0.94m.

**Table 6:** Selection of height percentile for different canopy densities.

Different canopy densities	D1	D2	D3	D4	D5
Detection height percentile	73.8%	69.0%	64.2%	59.5%	54.7%

However, the standard deviation of the detection distance tends to increase with decreasing density. It indicates that as the density of the canopy decreases, the canopy information detected becomes more unstable, which is consistent with the detection results under quasi-static conditions.

## 4 Discussion

The height monitoring and real-time attitude adjustment of the spray boom of the pesticide spraying machine are important ways for accurate and precise spraying, it is very important to maintain a reasonable and stable spray height between the nozzle and the spray target, and the parallel attitude between the spray rod and the ground. The automatic balance control technology of the boom is an important way to keep the relative attitude between the boom and the ground stable, and the monitoring of the boom tilt is the premise of the boom self adjustment. Ultrasonic sensor detection is an important way to obtain the height of the spray boom. In order to find out the influencing factors and laws of the real-time detection of the ultrasonic sensor, the detection experiments of steps, bare soil and wheat canopy were carried out. The influence of detection speed and distance on detection accuracy, the rule of burr signal in detection, and the influence of canopy sparsity on detection are analyzed.

### 4.1 Influence of detection distance and motion speed on detection accuracy

Ultrasound detection distance accuracy for regular step surfaces is high, with an average error of 1.35%. However, due to the influence of the detection range, the phenomenon of detecting two different distances of steps at the same time occurs at the steps. The height value of the detection is related to the ultrasonic intensity returned from the two step surfaces, so the detection range can cause detection advance or lag phenomenon. In the process of motion detection, there is a lag in the detection distance due to the existence of a certain period of the detection signal. The lagging distance is proportional to the velocity, the greater the velocity, the more pronounced the lag. The velocity factor also affects the hysteresis of the detection distance under bare ground, but at a lower slope of the bare ground. In other words, when the slope is small, the velocity-induced detection distance hysteresis is not significant, and when the detection distance fluctuates more, the velocity-induced detection distance hysteresis is significant. The phenomenon suggests that the factors influencing the detection distance lag are speed and slope, and that there is a coupling between the two. When the slope is gentle, the amount of advance or lag caused by the detection range is negligible. The same phenomenon exists for the detection of wheat canopy, i.e. the flat canopy in wheat fields is not affected by the delayed distance, but at the junction between the wheat canopy and the bare ground, a detection distance bias occurs. This conclusion is in common with the detection performance of LiDAR sensing (Dou et al. 2021a). A further improvement in control accuracy can be achieved by using this detection distance bias as a compensation for the delay time of the spray boom height system.

### 4.2 Regularity of burr signal

Regular step detection tests showed that a small amount of burr signal was present in the ultrasonic sensor for regular step detection. The maximum burr signal rate occurred in the quasi-static test at 0.05m/s with a burr rate of 0.22%. Burr signals were present in the field ground detection tests with a maximum burr signal rate of 2.15%. During the propagation of ultrasound, the surface of the obstacle encountered is rough, and the incident sound wave generates reflections in all directions, forming a scattering phenomenon. The scattering of sound waves is related to the shape of the obstacle. The soil slope is rough relative to the cardboard step surface and the sound waves are scattered on the surface of different blocks, resulting in instability and burr in the obtained ultrasound signal. Different leaf attitudes (spatial postures) result in different canopy structures. In the process of measuring the canopy using ultrasound, different echo signal waveforms were generated (Chen et al., 2020). During the detection process, the shaking of the wheat due to natural wind or mechanical influences causes the generation of the echo signal, which also produces a burr signal. However, the sparse nature of the acoustic waves as they propagate through the wheat canopy causes greater attenuation. In this study it was difficult to distinguish the burr signal from the non-canopy signal in the wheat canopy detection. In practice, oversized burr signals are clustered by Kmeans and no longer have an effect on the detection results, and then the appropriate height percentile is selected according to the different wheat sparsity levels as the basis for the spray boom height adjustment of the sprayer.

### 4.3 Influence of canopy sparsity on results

Wheat canopy detection tests showed that the detection bias of ultrasonic sensing became greater with decreasing wheat density. The wheat canopy is formed mainly by the natural curvature of the clumped leaves at the top to reflect acoustic waves. Therefore, wheat detection differs from other crops, such as blueberry detection systems where a minimum target of 12.5 mm is sufficient to detect the radial 19 mm radius radial foliage of wild blueberries. However, wheat plants typically have narrow leaves, thin spikes and a canopy where there is not enough density to reflect echoes, and ultrasonic sensors may not perceive weak echoes as valid signals (Yuan et al., 2019). Therefore, it is difficult for ultrasonic sensors to consistently detect effective signals reflected from wheat canopies. When the density is low, the returning ultrasonic echoes from the stems and leaves below the plant canopy result in a lower density of wheat. When the sound waves propagate beneath the wheat canopy, the irregular shape and distribution of the leaves causes some of the sound energy not to be reflected back, leading to scattering and absorption, resulting in unstable detection distances. The sparser the canopy the greater the detection error and the greater the error variance.

## 5 Conclusions

(1) A test platform with speed-adjustable ultrasonic sensors to detect the height of the spray boom was designed and a detection control system was built. Real-time detection height data acquisition and recording functions were implemented to provide a hardware basis for testing at different speeds and different detection targets.
(2) Ultrasonic sensor detection range calibration test results indicate that the sound cone is “spindle” type. In the range of 0.1-1.3m, the cone width increases linearly with increasing detection distance. In the detection distance of 1.3-1.7m range, the width of the sound cone changes more drastically and is not suitable for detection. The maximum cone width is 45.8cm and the maximum effective detection distance is 2.0m.
(3) Ultrasonic sensor has high accuracy in detecting the distance to the step surface under quasi-static conditions, and the average value of the detection error is 1.35%. With the increase of detection distance, affected by the detection radius and ultrasonic echo intensity, when the sensor detection distance is switched at the step, the actual offset from the step increases first and then tends to be stable with the increase of detection distance, and the maximum offset is 10.4cm. Because the sound cone of the ultrasonic sensor has a certain width, under the moving conditions, when the step surface changes or the ground slope is large, the speed is the main reason why the detection result of the sensor is ahead or behind the actual position of the step. When the terrain changes gently, the detection lead or lag caused by the detection range and speed can be ignored.
(4) Wheat canopy height detection test results suggest that, because the wheat canopy does not have a regular beam reflection plane, in the small strip of wheat canopy detection, the ultrasonic beam may detect the plant stalks and branches below the canopy, resulting in detection results are larger than the actual distance. The detection bias tends to increase as the density of wheat decreases, indicating that the lower the density of the canopy, the less ultrasound is reflected from the canopy and the more ultrasound is reflected from the stems and leaves below the canopy. The mean detection deviation of the ultrasonic sensors for the five wheat canopy densities was 0.14 m, and the maximum variance of the detection deviation was 0.07 cm2 when moving at a speed 0.3m/s-1.2 m/s. Therefore, K-means can be used as a clustering method for wheat canopy height data.

## Data availability statement

The original contributions presented in the study are included in the article/supplementary materials. Further inquiries can be directed to the corresponding authors.

## Author contributions

XZ performed most of the experiments with the assistance of CZ, SW, and HD. XZ, XW, and SY designed the software. CZ, LC, XZ, and XW designed the study, analyzed the data, and wrote the manuscript. All authors contributed to the study conception and design, read, and approved the final manuscript.

## Funding

This research was supported by National Modern Agricultural Industrial Technology System Project (CARS-03) and National Key Research and Development Program of China (2019YFE0125200) and Jiangsu Province Modern Agricultural Machinery Equipment and Technology Demonstration and Promotion Project (NJ2020-59) and Open Project of Intelligent Equipment Research Center, Beijing Academy of Agriculture and Forestry Sciences (KFZN2021W002).

## Conflict of interest

The authors declare that the research was conducted in the absence of any commercial or financial relationships that could be construed as a potential conflict of interest

## Publisher’s note

All claims expressed in this article are solely those of the authors and do not necessarily represent those of their affiliated organizations, or those of the publisher, the editors and the reviewers. Any product that may be evaluated in this article, or claim that may be made by its manufacturer, is not guaranteed or endorsed by the publisher.
